# Compartmentalized ocular lymphatic system mediates eye–brain immunity

**DOI:** 10.1038/s41586-024-07130-8

**Published:** 2024-02-28

**Authors:** Xiangyun Yin, Sophia Zhang, Ju Hyun Lee, Huiping Dong, George Mourgkos, Gordon Terwilliger, Aurora Kraus, Luiz Henrique Geraldo, Mathilde Poulet, Suzanne Fischer, Ting Zhou, Farrah Shalima Mohammed, Jiangbing Zhou, Yongfu Wang, Seth Malloy, Nicolas Rohner, Lokesh Sharma, Irene Salinas, Anne Eichmann, Jean-Leon Thomas, W. Mark Saltzman, Anita Huttner, Caroline Zeiss, Aaron Ring, Akiko Iwasaki, Eric Song

**Affiliations:** 1 Department of Ophthalmology and Visual Science, Yale School of Medicine, New Haven, CT USA; 2 Department of Immunobiology, Yale School of Medicine, New Haven, CT USA; 3 Department of Biomedical Engineering, Yale School of Engineering and Applied Science, New Haven, CT USA; 4 Section of Comparative Medicine, Yale School of Medicine, New Haven, CT USA; 5 Center of Evolutionary and Theoretical Immunology, Department of Biology, University of New Mexico, Albuquerque, NM USA; 6 Department of Internal Medicine, Cardiovascular Research Center, Yale School of Medicine, New Haven, CT USA; 7 Department of Cellular and Molecular Physiology, Yale School of Medicine, New Haven, CT USA; 8 Westlake Laboratory of Life Sciences and Biomedicine, School of Life Sciences, Westlake University, Hangzhou, China; 9 Department of Neurosurgery, Yale School of Medicine, New Haven, CT USA; 10 Stowers Institute for Medical Research, Kansas City, MO USA; 11 Section of Pulmonary and Critical Care and Sleep Medicine, Yale School of Medicine, New Haven, CT USA; 12 Université de Paris, INSERM, PARCC, Paris, France; 13 Department of Neurology, Yale School of Medicine, New Haven, CT USA; 14 Institut du Cerveau, Pitié-Salpêtrière Hospital, Centre National de la Recherche Scientifique, Institut National de la Santé et de la Recherche Médicale, Sorbonne Université, Paris, France; 15 Department of Chemical & Environmental Engineering, Yale School of Engineering and Applied Science, New Haven, CT USA; 16 Department of Dermatology, Yale School of Medicine, New Haven, CT USA; 17 Department of Pathology, Yale School of Medicine, New Haven, CT USA; 18 Department of Comparative Medicine, Yale School of Medicine, New Haven, CT USA; 19 Howard Hughes Medical Institute, Chevy Chase, MD USA

**Keywords:** Lymphatic vessels, Central nervous system

## Abstract

The eye, an anatomical extension of the central nervous system (CNS), exhibits many molecular and cellular parallels to the brain. Emerging research demonstrates that changes in the brain are often reflected in the eye, particularly in the retina^1^. Still, the possibility of an immunological nexus between the posterior eye and the rest of the CNS tissues remains unexplored. Here, studying immune responses to herpes simplex virus in the brain, we observed that intravitreal immunization protects mice against intracranial viral challenge. This protection extended to bacteria and even tumours, allowing therapeutic immune responses against glioblastoma through intravitreal immunization. We further show that the anterior and posterior compartments of the eye have distinct lymphatic drainage systems, with the latter draining to the deep cervical lymph nodes through lymphatic vasculature in the optic nerve sheath. This posterior lymphatic drainage, like that of meningeal lymphatics, could be modulated by the lymphatic stimulator VEGFC. Conversely, we show that inhibition of lymphatic signalling on the optic nerve could overcome a major limitation in gene therapy by diminishing the immune response to adeno-associated virus and ensuring continued efficacy after multiple doses. These results reveal a shared lymphatic circuit able to mount a unified immune response between the posterior eye and the brain, highlighting an understudied immunological feature of the eye and opening up the potential for new therapeutic strategies in ocular and CNS diseases.

## Abstract (web summary)

A study provides evidence for a shared lymphatic circuit that connects the posterior eye and the brain, allowing the generation of immune responses to protect the CNS against pathogens and tumours following intravitreal immunization.

## Main

**Fig. 1:**
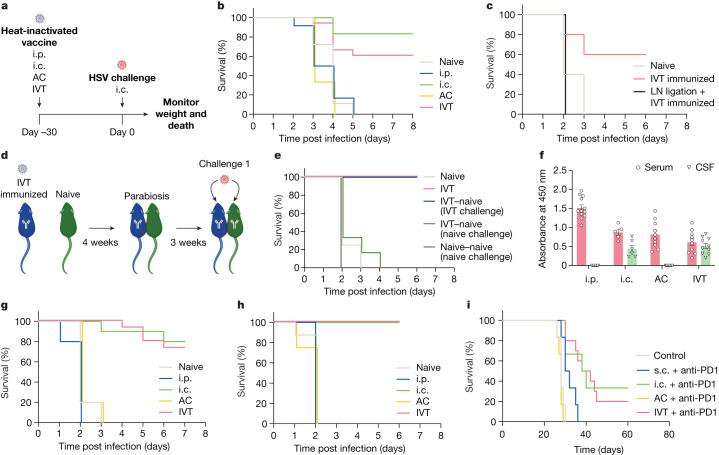
Antigens in the posterior eye elicit immune responses in the brain. **a**, Schematic of the schedule of procedures for the experiments described below. **b**, Wild-type C57BL/6J mice were immunized using heat-inactivated HSV-2 injection through i.p., i.c., AC and IVT administration. Survival was monitored after i.c. challenge with a lethal dose of HSV-2 30 days later (naive, *n* = 18; i.p., *n* = 12; i.c., *n* = 6; AC, *n* = 6; IVT, *n* = 18). **c**, dCLNs of mice were ligated using a cauterizer. Seven days later, mice were injected through the IVT route with heat-inactivated HSV-2. Their survival was monitored after i.c. challenge with a lethal dose of HSV-2 30 days later (naive, *n* = 5; IVT immunized, *n* = 5; LN ligation, *n* = 6). **d**, Schematic of the parabiosis mouse model and treatment plans. **e**, Mice were injected through the IVT route with heat-inactivated HSV-2. Four weeks later, the immunized mice were joined to naive mice. The immunized mice or naive mice were challenged through the i.c. route with a lethal dose of HSV-2 after 3 weeks, and their survival was monitored (naive, *n* = 4; IVT, *n* = 4; IVT–naive (IVT challenge), *n* = 6; IVT–naive (naive challenge), *n* = 2; naive–naive (naive challenge), *n* = 2). **f**, Anti-HSV-specific antibody was measured by enzyme-linked immunosorbent assay after different routes of HSV-2 immunization (i.p., *n* = 12; i.c., *n* = 6; AC, *n* = 10; IVT, *n* = 10). Data are shown as mean ± s.e.m. **g**, Wild-type C57BL/6J mice were injected with heat-inactivated HSV-1 through i.p., i.c., AC or IVT administration. Their survival was monitored after i.c. challenge with a lethal dose of HSV-1 30 days later (naive, *n* = 15; i.p., *n* = 6; i.c., *n* = 6; AC, *n* = 6; IVT, *n* = 18). **h**, As in **a**, but *S.* *pneumoniae* strain TIGR4 was used (naive, *n* = 8; i.p., *n* = 5; i.c., *n* = 5; AC, *n* = 8; IVT, *n* = 8). **i**, Mice were inoculated through the i.c. route with 50,000 GL261 luciferase-expressing (GL261–Luc) brain tumour cells, treated with irradiated GL261–Luc cells through s.c., i.c., AC or IVT administration (day 7) along with anti-PD1 (RMP1-14) antibodies (days 7, 9 and 11) and monitored for survival (naive, *n* = 6; s.c., *n* = 6; i.c., *n* = 6; AC, *n* = 6; IVT, *n* = 12). Data are representative of two independent experiments. The graphics in **a**,**d** were created with BioRender.com. Source Data

Herpes simplex virus (HSV) has the propensity to affect many tissues—including multiple neural tissues such as the dorsal root ganglia, the eyes and the brain—and is the most common cause of sporadic fatal encephalitis worldwide^2^. To understand protective immunity against HSV in the brain, we vaccinated mice with heat-inactivated HSV-2 through four administration routes—intraperitoneal (i.p.) to induce systemic immunity, intracranial (i.c.) to induce local brain immunity, intracameral (anterior chamber (AC)), and intravitreal (posterior chamber (IVT))—with the last two being ocular compartments that have unique anatomical connections to the nervous system (Fig. 1a). After a lethal i.c. HSV-2 challenge, all mice that underwent i.p. immunization succumbed to the infection, whereas i.c. immunization protected about 80% of the mice (Fig. 1b), showing that systemic immunity is not sufficient in providing protection against CNS infection. Remarkably, IVT immunization also protected the mice, but all AC-immunized mice, like i.p.-immunized mice, succumbed to the challenge (Fig. 1b), suggesting that IVT immunization drives a protective immune response in the brain.

**Extended Data Fig. 1:**
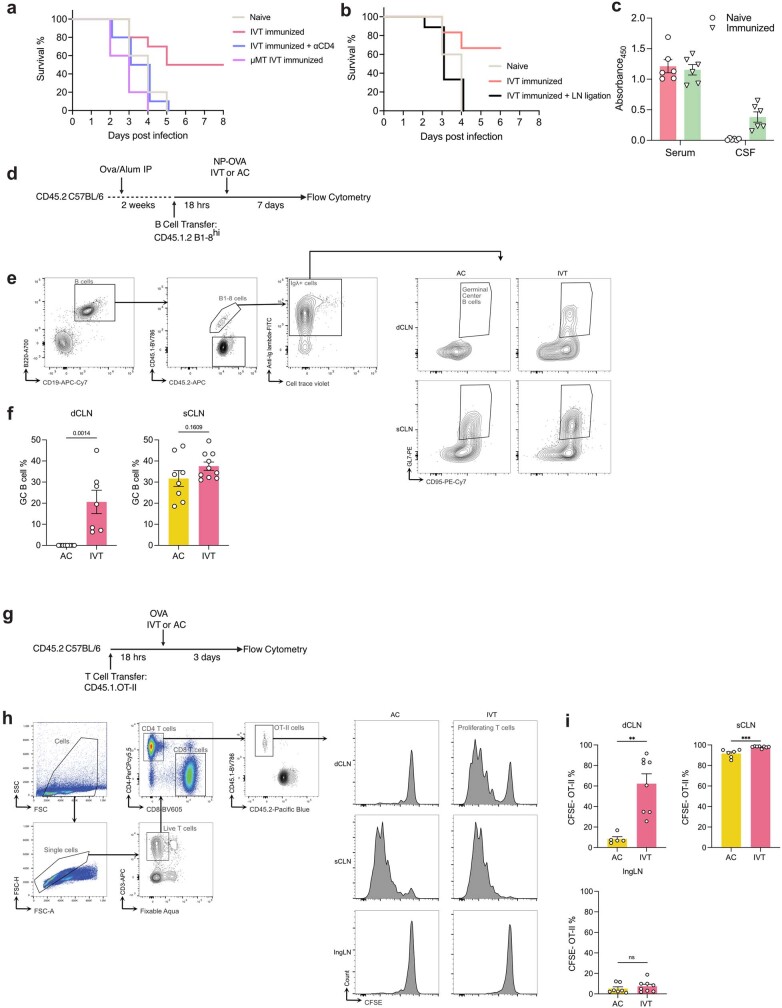
Immunization through the posterior compartment of the eye can induce an immunological protection in the brain. **a**, Similar to Fig. 1a, but B cell-deficient mice (μMT) were IVT injected with heat-inactivated HSV-2 and one group of WT IVT immunized mice were treated with CD4 depletion antibody before challenge. **b**, Similar to Fig. 1c, dCLNs were ligated after priming of the mice but before the intracranial rechallenge. Vaccination was completed first. Then, 7 days before the rechallenge, dCLNs were ligated, and mice survival was monitored after i.c. challenge with a lethal dose of HSV-2. (Naïve, n = 5; IVT immunized, n = 6; LN ligation, n = 9). **c**, Extending findings from Fig. 1d, the serum and CSF of both IVT immunized mice and naive mice were collected 3 weeks after parabiosis. Anti-HSV specific antibody was measured by ELISA (n = 6). **d**, Schematic of experimental plans. CD45.2 C57BL/6J recipient mice were primed by immunization. 2 weeks later, CD45.1.2 B1-8^hi^ B cells labelled with CellTrace™ Violet Cell Proliferation Kit were transferred intravenously into recipient mice. 18 h later, the mice were immunized with 10 μg NP–OVA though IVT or AC injection. Igλ^+^ light chain positive B1-8^hi^ cell proliferation and germinal center (GC) formation in dCLNs and sCLNs were analyzed at day 7. **e**, Flow cytometry gating strategy for B1-8^hi^ GC B cells. **f**, Quantification of B1-8 GC B cells in dCLNs and sCLNs (n = 7–10). **g**, Schematic of experimental plans. CD45.1 OVA specific CD4^+^ T cells labelled with CFSE were transferred intravenously into CD45.2 recipient mice. 18 h later, the mice were immunized with 50 μg OVA plus 1 μg Poly(I:C) though IVT or AC injection. FTY720 was i.p. injected to inhibit the circulation of primed T cells 24 h after immunization. The OVA-specific CD4^+^ T cell proliferation in dCLNs and sCLNs was analyzed 72 h after immunization. **h**, Flow cytometry gating strategy for analyzing OVA-specific CD4^+^ T proliferation (n = 5–8). **i**, Quantification of CFSE-negative OVA-specific CD4^+^ T cells in dCLNs, sCLNs, and ingLNs. Data shown as mean ± s.e.m. *P*-values were calculated using a two-tailed unpaired Student’s t-test. The graphics in **d** were created with BioRender.com. Source Data

As is the case for other organs, we reasoned that the immune response in the eye–brain axis must be mediated by local lymph nodes (LNs). To investigate the cellular mechanism facilitating the protection of the brain after IVT immunization, we first surgically ligated lymphatic vessels of the deep cervical LNs (dCLNs), which are critical to eliciting immune responses in the brain. This completely abrogated the protection provided by IVT immunization, indicating the importance of a local CNS–LN circuit that drains antigen to dCLNs (Fig. 1c). Additionally, as we ligated the lymphatic vasculature after priming but before the rechallenge, our results indicated that the lymphatic vessels of dCLNs were required to mount a recall response during the challenge (Extended Data Fig. 1b). The effector functions after HSV immunization are thought to be mediated by antiviral antibodies and CD4^+^ T cells^3^. Consistent with these reports, our observations showed that CD4^+^ T cell-depleted or B cell-deficient (μMT) mice were no longer protected after IVT immunization (Extended Data Fig. 1a). As efficient differentiation of follicular helper T cells and germinal centre B cells is required for the production of long-lived plasma cells and memory B cells that drive a superior humoral immune response following re-exposure to an antigen^4,5^, we quantified antigen-specific germinal centre B cell differentiation by adoptive transfer of B1-8^hi^ B cells^6^ after immunizing mice with NP–OVA through AC or IVT routes. Although both immunization routes can efficiently induce germinal centre B cell differentiation in superficial CLNs (sCLNs), only IVT immunization induces germinal centre B cell differentiation in dCLNs (Extended Data Fig. 1d–f). Similarly, IVT immunization significantly increases the level of antigen-specific CD4^+^ T cell proliferation in dCLNs compared to AC immunization (Extended Data Fig. 1g–i). These data indicate that the generation of localized antigen-specific CD4^+^ T and B cell responses in dCLNs by IVT immunization is required for host antiviral CNS protection.

To further address whether the IVT immunization-mediated protection was developed locally in the brain, we generated parabiotic mouse pairs with various immunization states (Fig. 1d). When IVT-immunized mice were paired with naive mice, only the mice that were initially immunized (left) and not their parabiotic partners (right) were protected against an i.c. challenge (Fig. 1e). Similarly, only the IVT-immunized mice, but not their naive parabiotic partners, had detectable levels of anti-HSV-2 antibodies in cerebrospinal fluid (CSF), which indicated that direct immunization is necessary to acquire anti-HSV-2 antibodies in CSF (Extended Data Fig. 1c). By contrast, because of the shared blood circulation, naive mice that were paired with IVT-immunized mice had a similar amount of anti-HSV-2 antibodies in their serum (Extended Data Fig. 1c). This suggested that serum antibodies do not mediate immune protection in the CNS; rather, immune protection occurs locally through the CSF. Furthermore, following examination of the localization of anti-HSV antibodies after different immunization routes, we found that, although all routes of immunization led to the presence of anti-HSV-2 antibodies in serum, only the i.c.- and IVT-immunized mice had detectable levels of anti-HSV-2 antibodies in CSF (Fig. 1f). Together, these data demonstrate that IVT immunization uniquely mediates CNS protection through a local antibody-dependent response.

**Extended Data Fig. 2:**
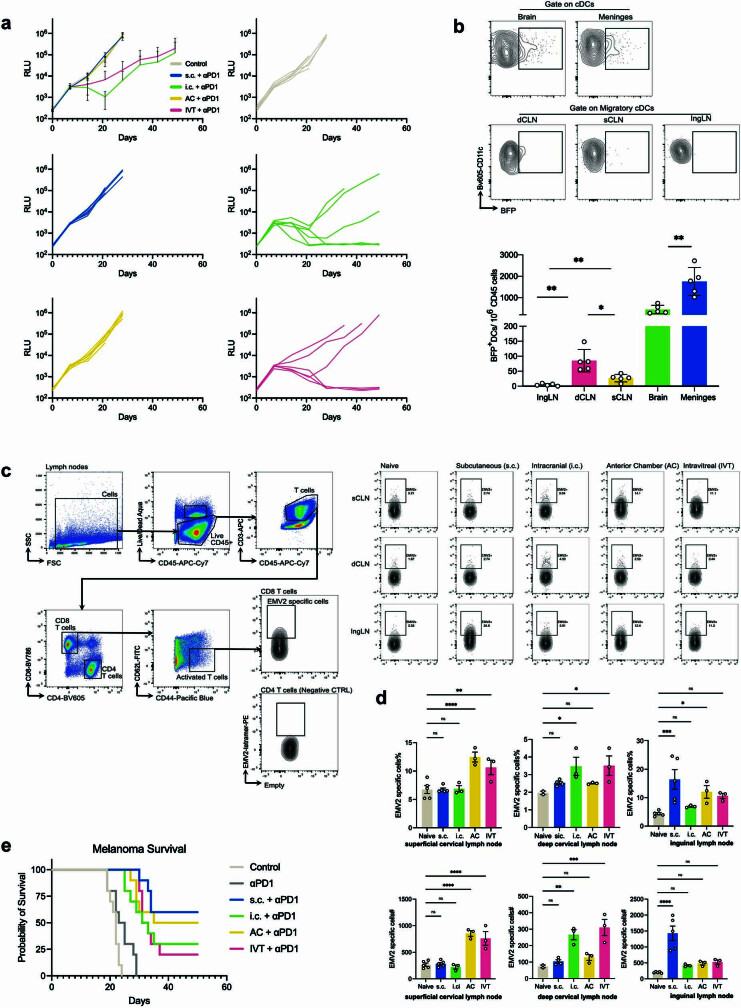
Immunization through the posterior compartment of the eye can provide therapeutic treatment of brain tumours. **a**, Mice were i.c. inoculated with 50,000 GL261-Luc cells and mice were treated with irradiated GL261-Luc through the routes of s.c., i.c., AC, or IVT administration (day 7) along with anti-PD-1 antibodies (days 7, 9 and 11) and monitored for tumour growth. RLU, relative luminescence units. (s.c., n = 6; i.c., n = 6; AC, n = 6; IVT, n = 6). **b**, Mice were i.c. inoculated with 50,000 CT2A-BFP cells. The percentage of BFP^+^ DCs in the tumour, ingLNs, meninges, dCLNs and sCLNs were analyzed 14 days after tumour injection (n = 5). **c**,**d**, Similar experiment as **a**. The percentage of EMV-2-specific CD8^+ ^T cells were analyzed in dCLN, sCLN and ingLN at day 14. **c**, Flow cytometry gating strategy for EMV-2-specific CD8^+ ^T cells. **d**, Quantification of EMV-2-specific CD8^+^ T cells in dCLNs, sCLNs and ingLNs. (Naïve n = 5; s.c., n = 5; i.c., n = 3; AC, n = 3; IVT, n = 3) **e**, Mice were s.c. inoculated with 50,000 B16 cells, treated with irradiated B16 cells through the routes of s.c., i.c., AC, IVT administration (day 7) along with anti-PD-1 antibodies (days 7, 9 and 11) and monitored for survival. (n = 10 for all groups). Data shown as mean ± s.e.m. *P*-values were calculated using a one-way ANOVA with multiple comparisons testing (Dunnett). Source Data

Finally, to determine whether this ocular–brain axis-mediated CNS protection extends beyond HSV-2, we examined the efficacy of a similar vaccination strategy against HSV-1 and *Streptococcus pneumoniae*, the main causative agent of bacterial meningitis^7,8^. Mice were immunized with heat-inactivated HSV-1 or *S.* *pneumoniae* through an i.p., i.c., AC or IVT route and rechallenged with a lethal dose of the same pathogen 30 days later. Similar to the results in the HSV-2 model, the data for both IVT- and i.c.-immunized mice showed them to have almost complete protection. By contrast, all of the naive, i.p.- and AC-immunized mice succumbed to the challenge (Fig. 1g,h). Even in a therapeutic setting in which an i.c. tumour was established before the administration of a cancer cell vaccine, immunization through the IVT or i.c. route resulted in superior protection compared to subcutaneous (s.c.) and AC immunization, which evoked only systemic immunity (Fig. 1i and Extended Data Fig. 2a). These data were consistent with previously described findings that IVT and i.c. immunization resulted in significantly greater proportions and numbers of antigen-specific CD8^+^ T cells compared to those following AC and s.c. immunization in dCLNs, but not in sCLNs or inguinal LNs^9^ (IngLNs; Extended Data Fig. 2b–d). By contrast, in a model of cutaneous melanoma, s.c. immunization showed better protection than IVT or i.c. immunization (Extended Data Fig. 2e), indicating that IVT and i.c. immunization induce a localized immune response but do not provide protection against peripheral tumours as well as a local immunization method does. Together, these data demonstrate that the ocular–brain immunological connection is specifically relevant and broadly applicable to many CNS diseases.

## The eye has a compartmentalized drainage system

Our immunization studies uncovered a divergent immune response between the AC and vitreous of the eye. It is thought that aqueous humour flows out from the AC through the conventional outflow pathway that includes the Schlemm’s canal and trabecular meshwork^10–12^. Additionally, a more recently appreciated unconventional route includes flow through the ciliary muscle and supraciliary and suprachoroidal spaces^13,14^. By contrast, the drainage from the posterior ocular compartment, particularly the vitreous, is unclear given the lymphatic vasculature in the eye and is still being explored^15,16^.

**Fig. 2:**
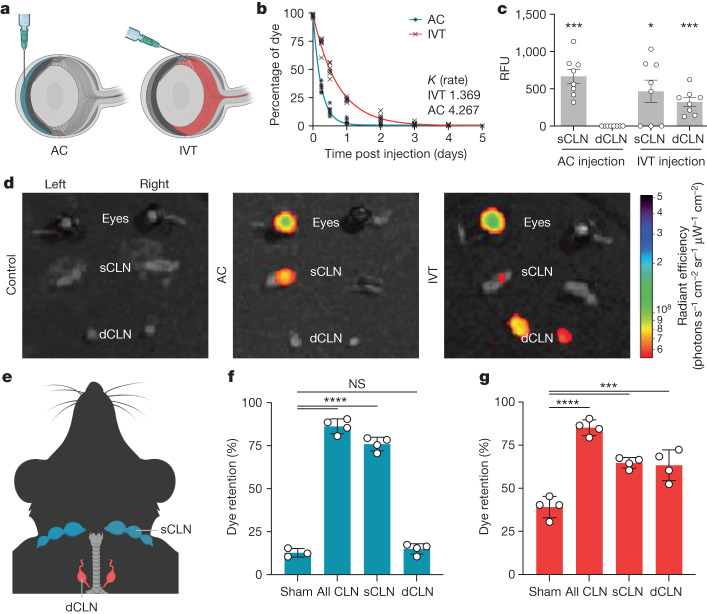
Eyes have a compartmentalized lymphatic drainage system. **a**, Schematic of AC and IVT injection of dye. **b**, C57BL/6J mice were injected with dye through the AC or IVT route. The percentage of dye retention in the eye was analysed from 6 h to day 5 post injection (AC, *n* = 6; IVT, *n* = 6). **c**, Dye was injected into the eye through the AC or IVT route. sCLNs and dCLNs were collected and measured using a fluorescence plate reader 1 h after injection. RFU, relative fluorescence unit (AC, *n* = 8; IVT, *n* = 8). Data are shown as mean ± s.e.m. ***P = 0.0002, AC sCLN; *P = 0.0167, IVT sCLN; ***P = 0.0009, IVT dCLN. **d**, Dye was injected into the left eye through the AC or IVT route, and eyes, sCLNs and dCLNs were collected for IVIS epifluorescence imaging. Representative background-subtracted heat maps of dye in the eye, sCLNs and dCLNs 1 h after injection are shown. **e**, Schematic of the anatomical locations of the sCLNs and dCLNs. **f**,**g**, sCLNs, dCLNs or both CLNs were surgically ligated. Two days later, dye was injected into the eye through the AC (**f**) or IVT (**g**) route. The percentage of dye retention in the eye was measured 12 h later. Data are shown as mean ± s.e.m. in **f** and **g** (sham AC, *n* = 3; *n* = 4 in all other conditions). ****P < 0.0001; ***P = 0.0002, IVT sCLN; ***P = 0.0003, IVT dCLN; NS, not significant. *P* values were calculated using a one-way analysis of variance (ANOVA) with multiple comparisons testing (Dunnett). The graphics in **a**,**e** were created with BioRender.com. Source Data

**Extended Data Fig. 3:**
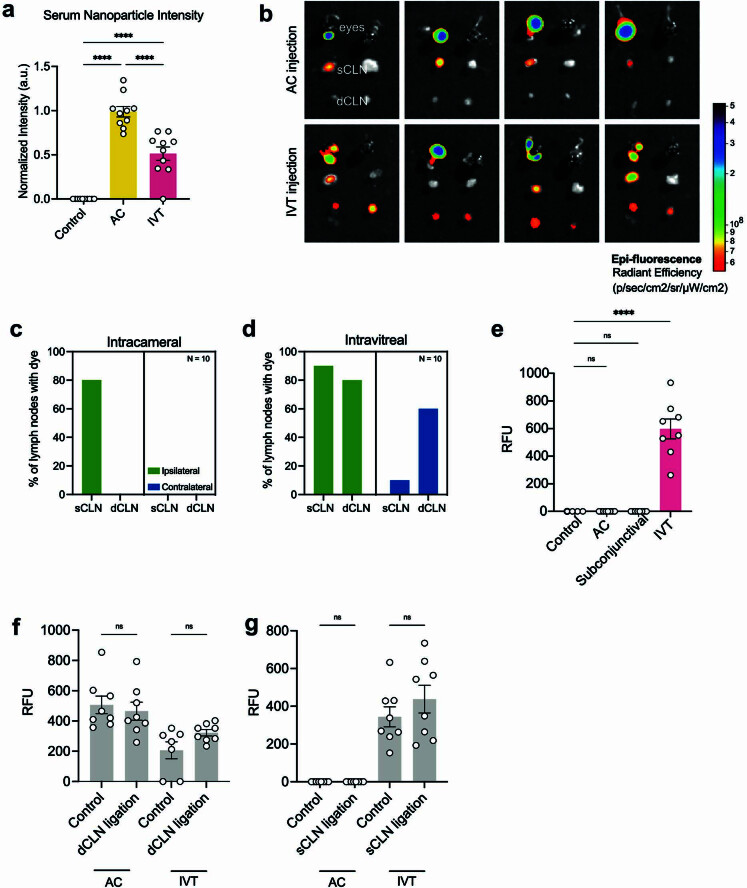
Eyes have a compartmentalized lymphatic drainage system. **a**, Nanoparticles were AC or IVT injected into the eye. Serum was collected, placed on a slide and imaged on a fluorescent microscope 1 h later after injection (n = 10). **b**, Dye was AC or IVT injected into the left eye. Eyes, sCLNs and dCLNs were collected for IVIS epifluorescence imaging. Representative background-subtracted heat maps of dye in the eye, sCLNs and dCLNs 1 h later after injection. **c**,**d**, Dye was AC (**c**) or IVT(**d**) injected into the eye and dye presence was measured using a fluorescent plate reader. The percentage of ipsilateral and contralateral LNs with dye was measured 12 h later (n = 10 mice). **e**, Dye was AC, IVT, or subconjunctivally injected into the left eye. dCLNs were collected and measured using a fluorescent plate reader 1 h later after injection (n = 8). **f**,**g**, dCLNs (**f**) or sCLNs (**g**) were surgically ligated. 2 days later, dye was AC or IVT injected into the eye. sCLNs (**f**) or dCLNs (**g**) were collected and measured using a fluorescent plate reader 1 h later after injection. RFU, relative fluorescence unit. (AC, n = 8; IVT, n = 8). *P*-values were calculated using a one-way ANOVA with multiple comparisons testing (Dunnett). Data shown as mean ± s.e.m. *P*-values were calculated using a one-way ANOVA with multiple comparisons testing (Dunnett) or two-tailed unpaired Student’s t-test. Source Data

To investigate the different drainage systems of the anterior and posterior compartments of the eye, we injected fluorescently labelled dextrans through AC or IVT routes and quantified the kinetics of dextran retention (Fig. 2a). The rate of dextran clearance in the eye after IVT administration (*K* = 1.369) was significantly slower than that after AC administration (*K* = 4.267; Fig. 2b). Dextrans with two different wavelengths of fluorescence and molecular weights were tested to ensure that this phenomenon was not dye specific. We reasoned that this observation could be explained by two possibilities; either the compartments have unique routes of drainage, or the rate-limiting step in the drainage of each compartment is different (that is, diffusion through the vitreous may be the slowest step). We reasoned that these potential explanations could be parsed out by observing dye localization in vivo. Using a previously described method^17^, we confirmed that both AC- and IVT-injected dye could be detected in the serum. However, significantly higher concentrations of dye were detected in the blood after AC injection compared to after IVT injection (Extended Data Fig. 3a). Whereas dye injected into the AC localized to the ipsilateral sCLNs, as previously reported^18–21^, IVT-injected dye localized to bilateral dCLNs in addition to the ipsilateral sCLNs (Fig. 2c–e and Extended Data Fig. 3b–d). As was observed after AC injection, dye does not drain to the dCLNs after subconjunctival injection (Extended Data Fig. 3e), indicating that penetration into the posterior eye or vitreous is required for drainage to the dCLNs. To test the requirement of these local lymphatic structures for drainage of the aqueous and vitreous humour, we surgically ligated the sCLNs, dCLNs or both. We first confirmed that ligation of dCLNs did not result in increased efflux into the sCLNs and vice versa (Extended Data Fig. 3f,g). Ligation of sCLNs, but not dCLNs, resulted in significantly increased dye retention in the eye following AC injection (Fig. 2f), consistent with the above observation. For IVT injection, either sCLN or dCLN ligation alone led to increased dye retention in the eye (Fig. 2g). Together, these results demonstrate a compartmentalized drainage system in the eye between the AC and the vitreous humour with a specific route from the vitreous humour to the dCLNs.

## Optic nerve sheath harbours functional lymphatics

**Fig. 3:**
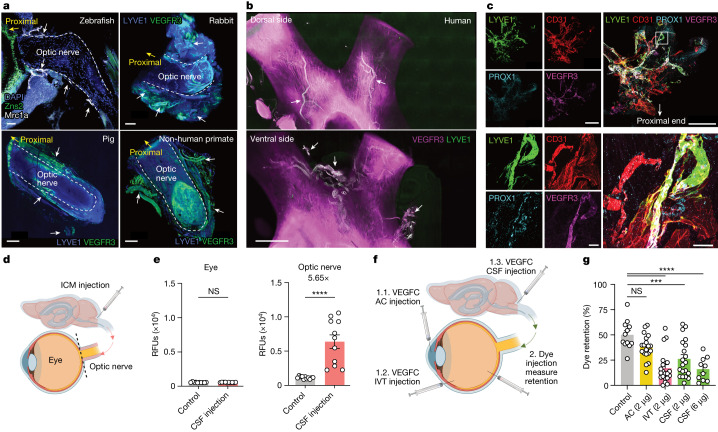
Optic nerve sheath lymphatics drain the posterior eye. **a**,**b**, Immunostaining of sections of optic nerve from zebrafish with lymphatics labelled for Mrc1a (white arrows; **a**, top left) and iDISCO immunolabelling of meningeal lymphatic vessels of rabbit (**a**, top right), pig (**a**, bottom left), non-human primate (**a**, bottom right) and human (**b**) optic nerve and chiasma, with lymphatics showing colocalization of LYVE1 and VEGFR3 (white arrows). DAPI, 4′,6-diamidino-2-phenylindole. **c**, Whole-mount wild-type mouse optic nerve sheaths stained for LYVE1, CD31, PROX1 and VEGFR3. The images at the bottom show a higher-magnification view of the area highlighted in the merged image at the top right. Scale bars, 50 μm (**a**, top left; **c**, bottom merged), 500 μm (**a**, top right; **c**, top merged), 1,000 μm (**a**, bottom left and right) and 3,000 μm (**b**). **d**, Schematic of ICM injection and the eye and optic nerve dissection for **e**. **e**, Dye was ICM injected, and fluorescence signal intensity was measured 1 h later in the eye and optic nerve (control, *n* = 11; CSF injection, *n* = 11). ****P < 0.0001. **f**, Schematic of injection methods for **g**. **g**, VEGFC was injected through the AC, IVT or ICM administration route. Two days later, dye was IVT injected into the eye, and the percentage of dye retention was measured 12 h after dye injection (control, *n* = 12; AC, *n* = 19; IVT, *n* = 20; CSF (2 μg), *n* = 20; CSF (6 μg), *n* = 10) ****P < 0.0001; ***P < 0.0002. Data are shown as mean ± s.e.m. *P* values were calculated using a one-way ANOVA with multiple comparisons testing (Dunnett) or two-tailed unpaired Student’s *t*-test. The graphics in **d**,**f** were created with BioRender.com. Source Data

**Extended Data Fig. 4:**
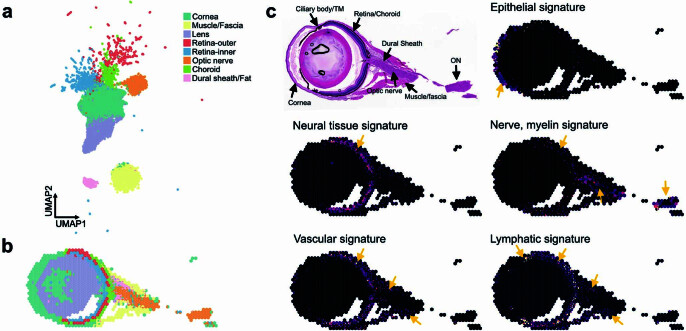
10x VISIUM Spatial sequencing of the mouse globe and optic nerve. Mouse eyes were processed using traditional FFPE-based histology preparation. Samples were processed using the 10x VISIUM platform and analyzed using Seurat. **a**, UMAP of different spatial sequencing blocks with categorization into overall tissue level structure. **b**, Projection of tissue-type clusters onto the globe and surrounding structures. **c**, Image of H&E staining and respective cell type gene signature projected onto spatial sequencing data (yellow arrows denote where the gene signature appears for given signature).

**Extended Data Fig. 5:**
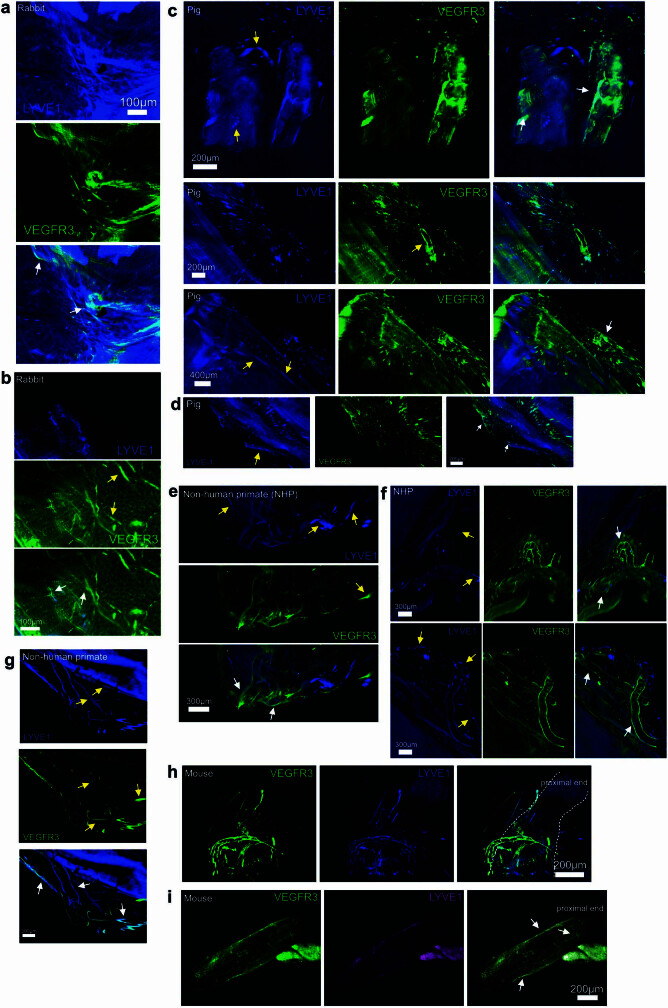
Anatomical characterization of meningeal lymphatic vessels covering optic nerve. Additional images of VEGFR3 and LYVE1 staining on optic nerves of rabbits (**a**,**b**), pigs (**c**,**d**), non-human primates (**e**,**f**,**g**), and mice (**h**,**i**). Yellow arrows indicate vascular structures with single staining for either LYVE1 or VEGFR3. White arrows indicate co-stained vascular structures.

Macromolecule localization into distinct LNs indicates that a lymphatic vessel network is probably mediating drainage from each of these compartments. Although a recent study described the presence of an ocular glymphatic system in mice, similar to that of the brain^15^, a functional network of lymphatic vessels mediating vitreous drainage has not been resolved. Therefore, we used a transcriptomic approach along with immunolabelling-enabled three-dimensional imaging of solvent-cleared organs (iDISCO)^22^ to obtain a global view of the lymphatic vascular network associated with the optic nerve in different vertebrate species. Spatial transcriptomics helped gather an organ-level view of lymphatic-like vasculature gene signatures in the eye and the optic nerve. In addition to the trabecular meshwork^10–12^ and retina, which have been found to harbour macrophages expressing some lymphatic-like gene signatures^23^, we found that the optic nerve sheath also contained lymphatic-like gene signatures (Extended Data Fig. 4). In parallel, iDISCO revealed lymph vessels covering zebrafish, mouse, rabbit, pig, non-human primate and human optic nerves (Fig. 3a,b and Extended Data Fig. 5), demonstrating an evolutionarily conserved feature of the lymphatic system. In all mammals, we saw vascular structures with co-localized staining of LYVE1 and vascular endothelial growth factor receptor 3 (VEGFR3; white arrows in Extended Data Fig. 5). We also saw vasculature stained for one but not the other, indicating the likely presence of LYVE1-negative collecting lymphatic vasculature^24^ or LYVE1-expressing macrophages (yellow arrows in Extended Data Fig. 5). Notably, the lymphatic vasculature was localized to membranes surrounding the nerve, not the nerve itself, in parallel to what is observed in the brain and the dural meninges^25,26^.

**Extended Data Fig. 6:**
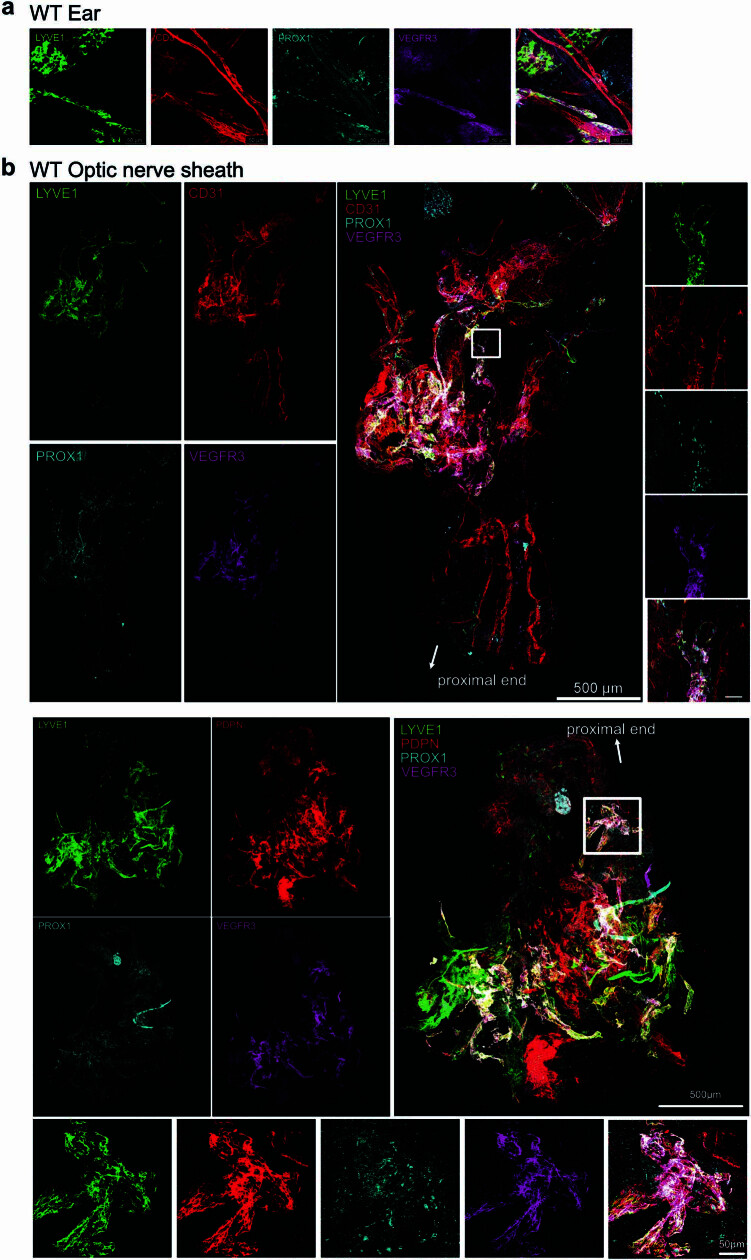
Detailed analysis of optic nerve sheath lymphatics. **a**, Whole-mount murine ventral ear sheet stained with LYVE1, CD31, PROX1 and VEGFR3. **b**, Whole-mount of optic nerve sheaths of WT mice. Top: LYVE1, CD31, PROX1 and VEGFR3 were co-stained. Bottom: LYVE1, PDPN, PROX1 and VEGFR3 were co-stained.

**Extended Data Fig. 7:**
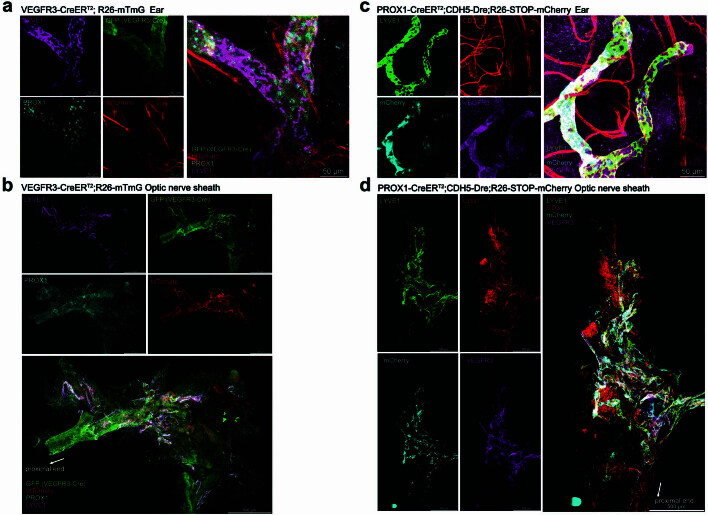
Detailed analysis of optic nerve sheath lymphatics with lymphatic reporter mice. **a**, **b**, Whole-mount murine ventral ear sheet and optic nerve sheaths of VEGFR3-CreER^T2^;R26- mTmG mice that were stained with LYVE1 and PROX1 (**a**, scale: 50 μm; **b**, scale: 500 μm). **c**, **d**, Whole-mount murine ventral ear sheet and optic nerve sheaths of PROX1-CreER^T2^;CDH5-Dre;R26-STOP-mCherry mice that were stained with LYVE1, CD31 and VEGFR3 (**c**, scale: 50 μm; **d**, scale: 500 μm).

To improve the sensitivity of the stains applied, we dissected out the optic nerve sheath completely from the optic nerve. Without perfusion fixation of mice, the optic nerve sheath retracted following enucleation, resulting in bunching of the lymphatic vasculature towards the proximal end of the nerve (Extended Data Fig. 5h). To address this, we carried out perfusion fixation before enucleation, preserving the anatomy of the lymphatic network along the nerve (Extended Data Fig. 5i). Using the well-characterized ear lymphatic system as a control (Extended Data Fig. 6a), we validated the presence of optic nerve sheath lymphatics using additional markers (VEGFR3, PROX1, LYVE1, CD31 and podoplanin; Fig. 3c and Extended Data Fig. 6b). To further characterize these vasculatures using a genetic approach, we used two different reporter mice enabling highly specific staining of lymphatics: VEGFR3-CreER^T2^;R26-mTmG mice, and PROX1-CreER^T2^;CDH5-Dre;R26-STOP-mCherry mice (Extended Data Fig. 7). Together, these parallel methods confirmed that lymphatics indeed exist in the optic nerve sheath.

**Extended Data Fig. 8:**
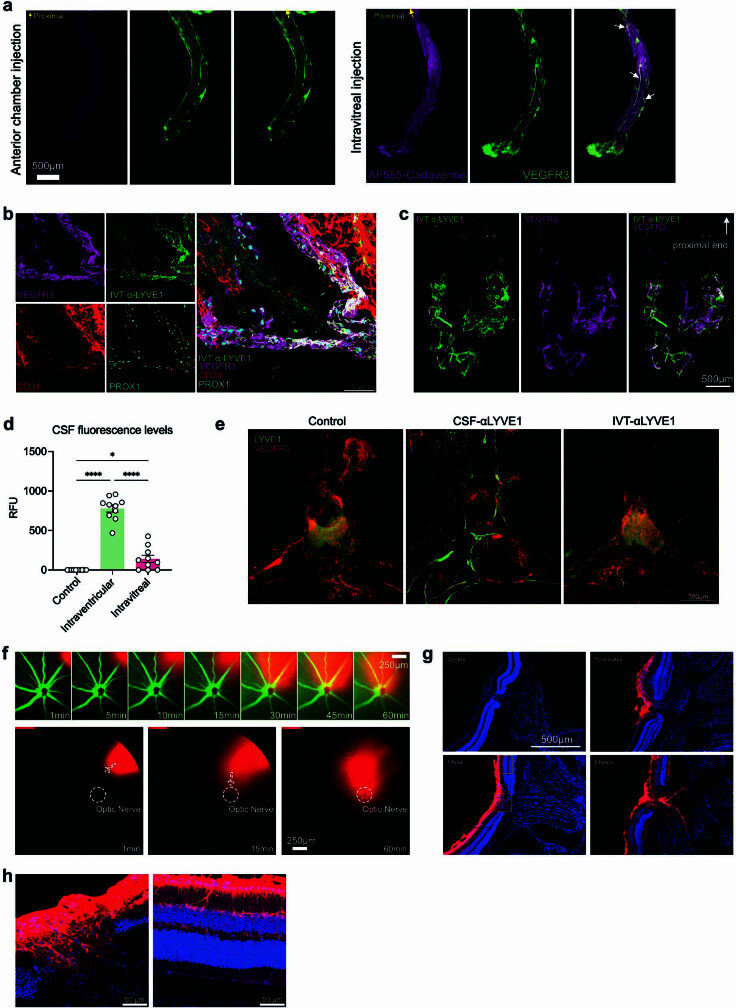
Optic nerve sheath lymphatics drain the posterior eye. **a**, Whole-mount murine optic nerve VEGFR3 staining and tracer signal on optic nerve 1 h later after AC (left) or IVT (right) injection. White arrowheads point to co-localization of VEGFR3 and tracer. **b**, **c**, Anti-LYVE1 antibody was IVT injected into the vitreous humor. Optic nerve sheaths were harvested 2 h later and co-stained with CD31, PROX1 and VEGFR3. **d**, Dye was IVT injected into the eye or intraventricularly into the brain. CSF was collected through the cisterna magna and measured using a fluorescent plate reader 1 h later after injection. **e**, Anti-LYVE1 antibody was injected IVT into the eye or ICM into the CSF. Dura was collected 2 h after injection and LYVE1 antibody binding on VEGFR3 positive lymphatic vessels was analyzed by immunofluorescence. Mice without anti-LYVE1 antibody injection were used as the control. **f**, AF647-OVA was IVT injected into the vitreous humor. Fluorescein was i.p. injected to label blood vessels. The kinetics of AF647-OVA drainage was tracked with Phoenix MICRON® IV imaging microscope. **g**, AF647-OVA was IVT injected into the vitreous humor. The eyes with optic nerve were harvested, and the location of AF647-OVA was analyzed at the indicated time points. **h**, Zoomed in images of **g**. Source Data

We then sought to evaluate the functionality of these lymphatics. Following IVT injection, but not AC injection, of a fluorescent tracer, the tracer was visualized in VEGFR3^+^ vasculature along the optic nerve (Extended Data Fig. 8a). Consistently, IVT-injected LYVE1 antibodies could effectively label optic nerve sheath lymphatics (Extended Data Fig. 8b,c), suggesting that macromolecules such as proteins can freely penetrate into these lymphatics from the vitreous. Our leading hypothesis of how molecules travel into these lymphatics was based on an understanding of the dural lymphatics of the brain^25,26^. We reasoned that molecules from the eye pass through the CSF into the optic nerve sheath lymphatics, similar to how molecules move from the brain to the dural lymphatics. To test this, we examined the distribution of dye and antibodies after IVT injection. We detected a trace amount of dye in the CSF after IVT injection compared to after brain intraventricular injection (Extended Data Fig. 8d). Furthermore, antibodies injected by the IVT route were not able to sufficiently label the dural lymphatics. These data indicated that IVT-injected dye probably entered the local CSF space around the optic nerve and did not drain to the dCLNs through previously characterized conventional brain dural meninges (Extended Data Fig. 8e). To parse out how macromolecules reach the lymphatic vasculature around the optic nerve sheath, we tracked how molecules injected into the vitreous left the eye in vivo. After IVT AF647-OVA injection, we observed that there was directional flow towards the optic nerve head (Extended Data Fig. 8f), consistent with previous findings that the pressure gradient between intraocular and i.c. spaces drives efflux of ocular fluid through the optic nerve^15^. Examination at a higher resolution after sectioning the eye revealed that the tracer was largely concentrated at the optic nerve head (Extended Data Fig. 8g), with some diffusion through the retinas. The diffusion through the retina showed a pattern similar to the previously described glymphatic patterns of the retinas, a paracellular perpendicular streak across the retina^15^ (Extended Data Fig. 8h).

**Extended Data Fig. 9:**
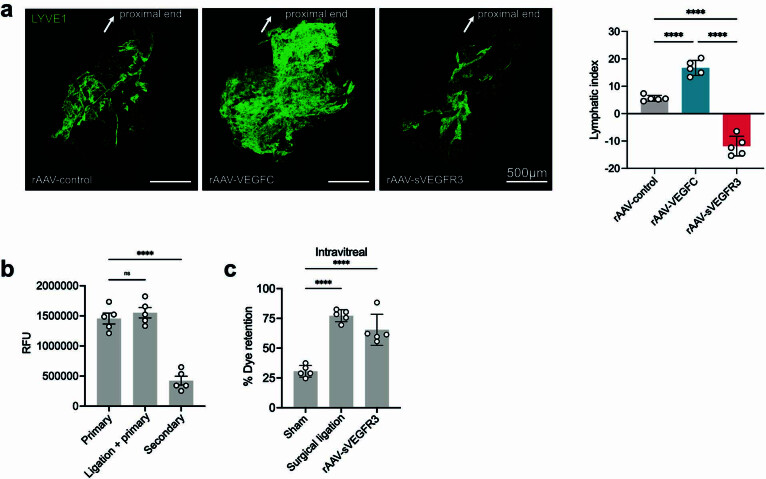
Lymphatic inhibition enables repeated rAAV administrations. **a**, Mice were IVT injected with rAAV-VEGFC, rAAV-sVEGFR3 or rAAV-control. Two months later, their optic nerve sheaths were harvested and stained with LYVE1. **b**, Similar to Fig. 4c, dCLNs were ligated before the primary rAAV-RFP injection. Their RFP intensity were quantified. **c**, Percentage of dye retention in the eye was measured for control, dCLN-ligated, or rAAV-sVEGFR3-injected mice. Data shown as mean ± s.e.m. *P*-values were calculated using a one-way ANOVA with multiple comparisons testing (Dunnett). Source Data

To validate the ability of these lymphatics to drain the vitreous, we delivered VEGFC, a lymphatic stimulator^27,28^, through three different compartmentalized routes: AC (targeting lymphatics of the anterior eye), IVT (targeting the posterior eye, optic nerve and possibly the anterior eye) or intracisterna magna (ICM; targeting optic nerve). First, using fluorescent tracers, we confirmed that ICM-injected tracers had access only to the optic nerve and not to the eye^15,29^ (Fig. 3d,e). Two days after VEGFC delivery, fluorescent dextrans were injected by the IVT route, and retention was analysed 12 h later (Fig. 3f). AC administration of VEGFC did not change dextran drainage from the vitreous, indicating that the anterior lymphatic stimulation is not sufficient in driving vitreous drainage. By contrast, IVT and ICM administration of VEGFC increased dextran drainage from the vitreous, the latter in a dose-dependent manner (Fig. 3g), supporting the observation that the posterior compartment of the eye drains to the dCLNs through lymphatic vasculature on the optic nerve sheath. To further confirm the role of VEGFC in modulating optic nerve sheath lymphatics, we also delivered recombinant adeno-associated virus (rAAV)-VEGFC or soluble VEGFR3 (sVEGFR3) through the IVT route and found that expression of VEGFC or sVEGFR3 resulted in significant increases or decreases in optic nerve sheath lymphatic vasculature, respectively (Extended Data Fig. 9a). In summary, these data highlight the existence of a functional lymphatic vessel network on the optic nerve sheath that connects the posterior eye to a set of LNs unique from the rest of the eye but shared with the rest of the CNS.

## Enhancing gene therapy via ocular lymphatics

**Fig. 4:**
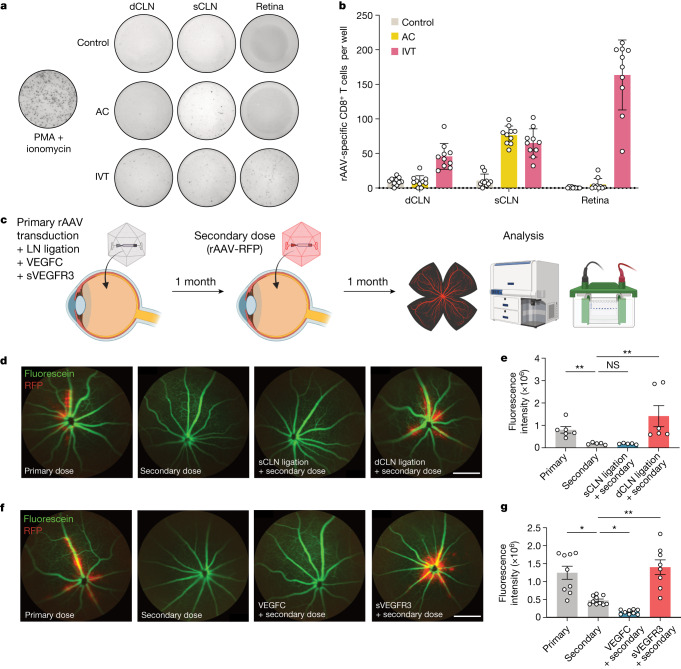
Lymphatic inhibition enables repeat rAAV administration. **a**,**b**, Mice were injected with rAAV-RFP through the IVT or AC route. Their dCLNs, sCLNs and retinas were collected 10 days later, and rAAV-specific immune responses were quantified using an ELISpot assay. For **c**–**g**, C57BL/6J mice were IVT injected with rAAV, and, 1 month later, were rechallenged with rAAV-RFP. The efficiency of rAAV-RFP transduction was analysed by imaging 1 month later. **c**, Schematic of experimental plans. **d**, In vivo fluorescence fundus imaging to visualize vessels (green) and RFP transduction (red) in different LN ligation conditions. Scale bar, 500 μm. **e**, Quantification of RFP intensity from **d** (primary, *n* = 6; secondary, *n* = 5; sCLN ligation, *n* = 5; dCLN ligation, *n* = 6). **P = 0.0095, primary versus secondary; **P = 0.0044, secondary versus dCLN ligation. **f**, In vivo fluorescence fundus imaging to visualize vessels (green) and RFP transduction (red) with addition of VEGFC or sVEGFR3. Scale bar, 500 μm. **g**, Quantification of RFP intensity from **f** (primary, *n* = 9; secondary, *n* = 10; VEGFC, *n* = 9; sVEGFR3, *n* = 8). *P = 0.0167, primary versus secondary; *P = 0.0334, secondary versus VEGFC; **P = 0.0096, secondary versus sVEGFR3. *P* values were calculated using a one-way ANOVA with multiple comparisons testing (Dunnett). Data are shown as mean ± s.e.m in **b**, **e** and **g**. The graphics in **c** were created with BioRender.com. Source Data

**Extended Data Fig. 10:**
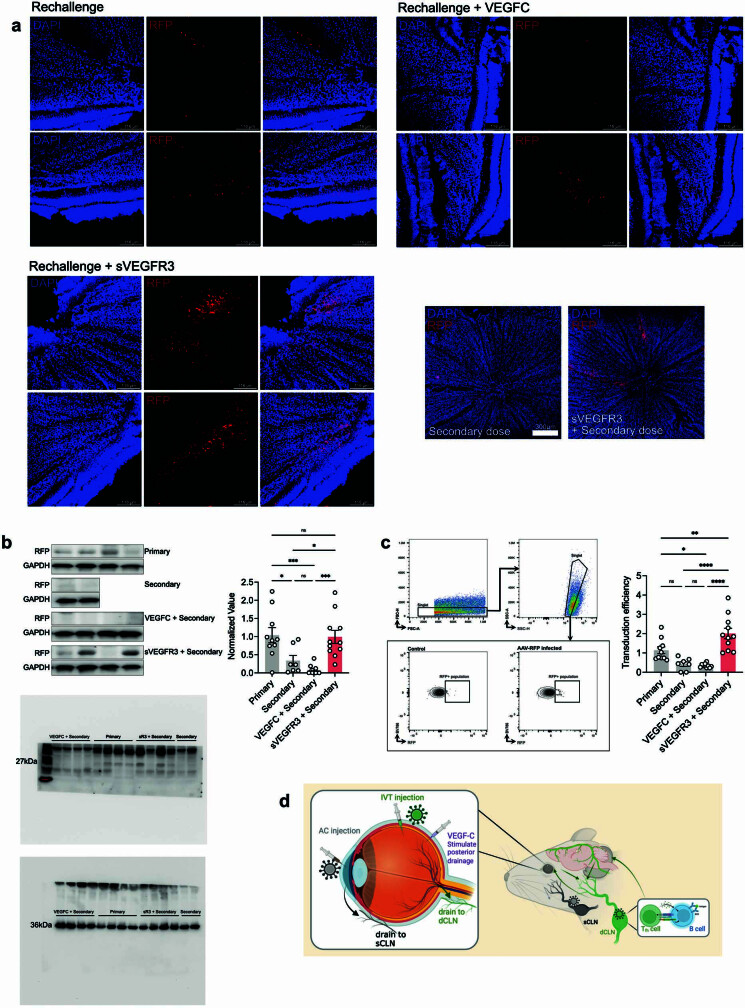
Lymphatic inhibition enables repeated rAAV transduction. **a**, Whole mount confocal images of rAAV-RFP rechallenge in murine retinas after treatment with VEGFC or sVEGFR3 during primary infection. **b**, Similar to Fig. 4c, murine retinas were collected, and RFP expression was quantified using western blot. **c**, Similar to Fig. 4c, murine retinas were collected, and RFP expression was quantified using flow cytometry. **d**, Schematic summary of findings. The graphics in **d** were created with BioRender.com. Source Data

Having confirmed the immunological importance of this posterior ocular drainage, we next investigated potential therapeutic applications that harness this finding. rAAVs are the most common in vivo gene delivery vectors owing to their safe and effective transduction^30^. Additionally, using AAV2 as a vector to treat patients with RPE65-mediated inherited retinal dystrophy was the first gene therapy approved by the US Food and Drug Administration^31^. However, despite its modified immunological properties, acquired immunity to rAAV limits the efficacy of its repeated administration^32,33^. We first characterized the rAAV-specific CD8^+^ T cell induction in LNs and retinas after AC and IVT administration of rAAV using an ELISpot assay. Although both AC and IVT injection induced a similar amount of rAAV-specific CD8^+^ T cells in the sCLNs, IVT injection specifically induced more rAAV-specific CD8^+^ T cells in the dCLNs and the retinas, which were not observed after the AC injection (Fig. 4a,b). We reasoned that lymphatic drainage on the optic nerve contributes to the immunological response to rAAV, especially for retina-based therapeutic approaches, thereby limiting its utility. We examined whether ablation of ocular lymphatic vessels could enable prolonged therapeutic efficacy after repeated rAAV injection into the eye. To test this, we first administered the primary dose of rAAV and, 1 month later, administered a second dose of RFP-expressing rAAV (rAAV-RFP). As a baseline control, naive mice were given rAAV-RFP at the time of the second dose (Fig. 4c). Similar to the findings of previous reports and consistent with our observation regarding local CNS immunity, the level of RFP expression after the secondary infection was significantly lower than that after the primary infection (Fig. 4d,e). The importance of ocular lymphatic vessels was confirmed by carrying out a similar experiment following either sCLN or dCLN ligation, which demonstrated that only dCLN ligation was sufficient in mitigating the immune response to rAAV and recovering secondary rAAV-RFP transduction efficiency (Fig. 4d,e). Additionally, we confirmed that the ligation procedure did not affect the primary challenge (Extended Data Fig. 9b). As surgically ligating the LNs would not be a therapeutically viable option for treatment, we used an alternative method to inhibit the molecular activity of lymphatics: IVT administration of sVEGFR3, which we found to inhibit lymphatic drainage to a similar extent as surgical ligation (Extended Data Fig. 9c). We saw that immune response to rAAV was further exacerbated by the presence of VEGFC, which is known to increase immune responses by stimulating lymphatic drainage^9,28^. Conversely, inhibiting VEGFC signalling during the primary rAAV transduction using sVEGFR3 allowed for the second dose to be just as effective as the primary infection (Fig. 4f,g and Extended Data Fig. 10a–c). These experiments establish the role of posterior ocular lymphatics in modulating the efficacy of rAAV-based gene therapies and suggest a platform to overcome current barriers limiting repeated gene therapy.

## Discussion

We have demonstrated that the posterior compartment of the eye has a unique lymphatic drainage system that anatomically engages with the CNS meningeal lymphatic network at the dCLNs. Previous studies have described the existence of sparse lymphatic capillaries on human and non-human primate optic nerves^34,35^; using spatial transcriptomics, several imaging techniques, genetic manipulation in vivo and tracer-injection experiments, we mapped the larger lymphatic vasculature network in the optic nerve sheath and described its functionality in draining the vitreous to the dCLNs. These results are in accordance with the known nexus between the eye and CNS^1^ and further extend this concept by demonstrating a shared immune surveillance mechanism between the two sites (Extended Data Fig. 10d).

Previously, both the eye and brain, which are devoid of traditional lymphatic vessels, were considered immune-privileged tissues because foreign tissue grafts can survive after implantation^36,37^. Furthermore, antigens delivered to both the anterior and posterior chamber of the eye have been reported to induce peripheral immune tolerance^18,38–40^. However, our study revealed that pathogenic antigens delivered to the posterior eye through IVT vaccination can drain to dCLNs and initiate a local protective immune response in the brain that is dependent on CD4^+^ T cells, B cells and local antibodies in the CNS compartment. Although the use of different disease models and therapeutic interventions in our studies shows the universality of our findings, it is likely that different pathogens or diseases may skew towards specific arms of the adaptive immune response. Of note, in our experiments and those of others, it is not that the sCLNs lack antigens from the CNS; rather drainage into the sCLNs is not sufficient to mount an immune response in the CNS, and future studies uncovering the fundamental difference between these two LNs may give us better insight into the observations described here. One possibility is that sCLNs and dCLNs may differ in their ability to generate effective brain-homing T cells or brain-resident T cells, which are reported to provide protection against pathogen reinfection in the brain^41,42^. Besides the protective role of local antibodies in IVT vaccination, it will be important to investigate whether brain-resident T cells play a role during challenge.

In addition, the mechanisms that guide these immune responses to the brain still need to be further investigated. The present findings imply that immune tolerance to foreign antigens in the posterior eye and brain is driven by immune suppression, both passive and active, and not due to a lack of an immune response. Thus, the posterior drainage system of the eye may play a pivotal role in modulating immune activation and suppression for various diseases involving the CNS. Current conventional methods to treat or diagnose diseases of the CNS are either invasive or fail to initiate local immunity. In cases with a suspected increased mass effect in the CNS due to infection or malignancy, it can often be dangerous to access the CSF through methods such as a lumbar puncture. Although far from clinical applicability, our studies propose the vitreous as a relatively non-invasive and more accessible route to elicit CNS immunity.

We use these concepts to illustrate that disruption of the posterior lymphatic vessels can negate immune response to rAAV delivered to the posterior eye and thus restore therapeutic benefit. Contrary to the notion of immune privilege, rAAV therapy for ocular diseases faces substantial hurdles due to the cellular and humoral immune response that it initiates^33^. These findings further confirm that this optic nerve drainage system identified in our study is responsible for the immunological outcome in response to antigens in the vitreous humour. The results also signify that the posterior lymphatic drainage system of the eye is a versatile and accessible target for manipulating immune responses in many different contexts.

Beyond the immunological implications of the posterior drainage system, how the mechanics of the posterior drainage pathway can be harnessed to treat ocular diseases merits further study. Specifically, the identification of a posterior eye lymphatic drainage system provides a potential efficient drainage route to modulate intraocular pressure or remove unwanted fluid in disease states such as glaucoma or macular oedema, respectively. Also, with the recently described presence of the glymphatic system in the eye^15^, more work is required to elucidate how these pathways interact with each other to provide homoeostasis in the eye, similar to in the brain. Together, the findings of the present study establish that the connection between the posterior eye and the brain is not only a neuronal one but also a shared immune circuit that coordinates immunity from the periphery to the CNS.

Why this compartmentalized lymphatic drainage system in the eye has evolved poses an interesting question. One possibility is that the centralization of the CNS and the emergence of lymphatic networks in vertebrates drove the polarization of the two lymphatic drainage systems in the eye. A clue to this may be in the lateralization that we observe during drainage to the sCLNs but not dCLNs, which is probably a consequence of the anatomical separation of skin, muscle and tissue surrounding the orbit. Contrarily, bilateral drainage to the dCLNs from the posterior eye occurs through a network of CSF-draining lymphatics. The anterior lymphatics act in synchrony with the cornea and conjunctiva, a generalized mucosal surface with constant exposure to outside invaders that may never have access to the brain. The optic nerve lymphatics we describe here connect the posterior eye (retina) with the brain, allowing coordinated immune responses that may prepare the brain for imminent threats. The segregation of eye immune responses into anterior and posterior lymphatic networks may predate mammals, and future studies in other vertebrates may help illuminate when and why this innovation first emerged.

## Methods

### Mice

Six- to ten-week-old mixed-sex C57BL/6J, B6.Cg-Tg(TcraTcrb)425Cbn/J (OT-II), B6.129P2(C)-Ightm2Cgn/J (B1-8) and B6.129S2-IghtmICgn/J (μMT) mice were purchased from Jackson Laboratory and Charles River and subsequently bred and housed at Yale University. PROX1-CreER^T2^;CDH5-Dre;R26-STOP-mCherry and VEGFR3-CreER^T2^;R26-mTmG mice were gifts from the laboratory of J.-L.T. All procedures used in this study complied with federal guidelines and the institutional policies of the Yale School of Medicine Animal Care and Use Committee. Age- and sex-matched animals were randomly assigned to control or treatment groups in each experiment. No statistical methods were used to predetermine sample sizes. Sample sizes were empirically determined based on previously published studies and to ensure sufficient statistical power. Investigators were not blinded to experimental groups, as measurements were not subjective.

### Cells

GL261–Luc cells were a gift from J. Zhou (Yale Neurosurgery) and were cultured in RPMI supplemented with 10% FBS, 1% penicillin–streptomycin and 1% sodium pyruvate. CT2A–BFP cells were a gift from T. Mathivet (Paris Centre de Recherche Cardiovasculaire). B16 cells were a gift from N. Palm (Yale Immunobiology). Cells were negative for mycoplasma contamination.

### Bacteria and viruses

The HSV-1 KOS strain and HSV-2 strains 186syn^−^TK^−^ and 186syn^+^ were gifts from D. Knipe (Harvard Medical School). These viruses were propagated and titrated on Vero cells (ATCC CCL-81) as previously described^43^. Vero cells were negative for mycoplasma contamination.

*S.* *pneumoniae* (ATCC 6303) was grown on 10% sheep blood agar plates (BD Biosciences) overnight at 37 °C (5% CO_2_). These colonies were then transferred to Todd Hewitt broth and grown overnight. The number of bacteria was enumerated using the optical density at 600 nm.

### Optic nerve tissues and fluorescence immunocytochemistry

*Tg(mrc1a:eGFP)*^44^ adult male and female 1-year-old zebrafish (*n* = 3) were provided by B.W. Weinstein at the US National Institutes of Health (Bethesda, MD). Zebrafish were euthanized by an overdose of MS-222 and whole heads were excised and fixed in 60 mM HEPES-buffered 4% PFA (pH 7.4) overnight at room temperature. Samples were then decalcified in 10% w/v EDTA solution (pH 7.4) for 5 days at room temperature. Heads were then snap frozen in OCT and 30-μm-thick cryosections were obtained. Cryosections containing the optic nerve were stained with the primary antibodies mouse anti-zebrafish Zns2 (ZIRC, ZDB-ATB-081002-34; 1:50) and chicken anti-GFP (Aves, catalogue no. GFP-1010; 1:500) followed by the secondary antibodies AF488-conjugated goat anti-mouse IgG (Jackson Immuno 115-545-146) and Cy5-conjugated donkey anti-chicken IgY (Jackson Immuno 703-175-155) with DAPI (5 μg ml^−1^) for nuclear staining. Images were acquired in a Zeiss LSM.

Eyes from rabbits and pigs were collected post-mortem from animals euthanized owing to unrelated health conditions. Eyes were removed within an hour of death and immersion fixed in 10% neutral-buffered formalin. Intact eyes from two female rhesus macaques (*Macaca mulatta*), between 25–28 years of age and euthanized owing to unrelated health conditions were collected post-mortem. Human optic nerves and chiasmas were obtained through the Yale Pathology Tissue Services through the Tissue Procurement and Distribution service. A mini standard operating procedure was written and approved for these samples. Eyes were removed within an hour of death and immersion fixed in 10% neutral-buffered formalin. Optic nerves with meningeal sheaths were cut and processed through the iDISCO protocol below.

Mouse optic nerve sheaths were dissected after mice were anaesthetized and transcardially perfused with cold PBS and 4% PFA (Sigma-Aldrich) sequentially. Mice were first decapitated, and the skull was exposed by cutting off the skin and scalp. One midline cut down the skull and two transverse cuts on both sides of the skull were made. Tweezers were used to peel and remove both halves of the skull to expose the brain. The brain was lifted from the posterior, and vasculature connecting the brain to other parts of the head was cut until the brain could be lifted enough to expose the optic tract. Brain tissue was removed after cutting just anteriorly to the optic chiasma. The extracranial optic canal was unroofed by removing parts of the skull above the eyes to expose the intracanalicular and canal segments of the optic nerve. Tissue surrounding the optic nerve was carefully dissected to free the nerve, and the eye was cut off at the optic nerve head. After removing the optic nerve, the optic nerve sheath was cut along the length of the nerve and removed for staining and whole-mount imaging.

The PROX1-CreER^T2^;CDH5-Dre;R26-STOP-mCherry and VEGFR3-CreER^T2^; R26-mTmG mice were injected through the i.p. route with 100 μl tamoxifen (10 mg ml^−1^; Sigma-Aldrich, T5648) for 5 consecutive days and optic nerve sheaths were collected 2 days later as above. Then optic nerve sheaths were fixed in 1% PFA for 1 h and immediately processed in a blocking solution (10% normal donkey serum, 1% bovine serum albumin, 0.3% PBS–Triton X-100) for 1 h at room temperature. For detection of lymphatic vessels, samples were incubated with primary antibodies overnight at 4 °C, and then washed five times at room temperature in PBS with 0.5% Triton X-100, before incubation with fluorescence-conjugated secondary antibodies diluted in PBS with 5% normal donkey serum. Lymphatic vessel images were acquired using a Leica confocal microscope (Stellaris 8). The following antibodies were used: goat anti-mouse VEGFR3 (R&D, No. AF743, 1:400), rat anti-mouse LYVE1 (R&D, No. MAB2125, 1:400), Syrian hamster anti-mouse podoplanin (BioLegend,127402, 1:500), rabbit anti-Prox1 (Angiobio, 11-002 P, 1:200), Armenian hamster anti-mouse CD31 (Gene Tex, 2H8, 1:1,000). The primary antibodies were detected with appropriate AF405-, AF488-, AF555- and AF647-conjugated secondary antibodies (Thermo Fisher, 1:500) after 2 h of incubation at room temperature. ProLong Gold Antifade Mountant (Invitrogen, P36930) was used for mounting the sections.

### Antibodies for flow cytometry

Anti-CD3 (145-2C11, APC, 152306, 1:200), anti-CD4 (RM4-5, PerCP, 100538, 1:400; RM4-5, BV605, 100548, 1:400), anti-CD8α (53-6.7, BV605, 100744, 1:400; 53-6.7, BV785, 100750, 1:400), anti-CD11b (M1/70, BV711, 101242, 1:500), anti-CD19 (6D5, APC–Cy7, 115530, 1:400), anti-IA and IE (M5/114.15.2, AF488, 107616, 1:800), anti-CD44 (IM7, AF700, 103026, 1:200; BV421, 103040, 1:200), anti-CD45 (30-F11, APC–Cy7, 103116, 1:200), anti-CD45.1 (A20, BV785, 110743, 1:300), anti-CD45.2 (104, Pacific Blue, 109820, 1:200), anti-CD64 (X54-5/7.1, PE, 139304, 1:300), anti-CD95 (Jo2, PE–Cy7, 557653, 1:400), anti-B220 (Ra3-6B2, AF700, 103232, 1:400), anti-GL7 (GL7, fluorescein isothiocyanate (FITC), 144603, 1:400), anti-NK1.1 (PK136, APC–Cy7, 108724, 1:400), anti-TCRβ (H57-597, APC–Cy7, 109220,1:200) were purchased from BD Biosciences or BioLegend. Anti-Igλ light chain, (JC5-1, FITC, 130-098-415, 1:400) was purchased from Miltenyi Biotec.

### Isolation of mononuclear cells

For brain tissues, tissues were collected and incubated in a digestion cocktail containing 1 mg ml^−1^ collagenase D (Roche) and 30 μg ml^−1^ DNase I (Sigma-Aldrich) in RPMI at 37 °C for 45 min. Tissues were pipetted to break tissue down and filtered through a 70-μm filter. Then, cells were mixed in 3 ml of 25% Percoll (Sigma-Aldrich) solution and centrifuged at 580*g* for 15 min without brake. The Percoll layer was removed, and cell pellets were treated with 0.5 ml ACK buffer and spun for 5 min at 500*g*. Then, the cell pellets were resuspended in FACS buffer (PBS + 2% FBS + 1 mM EDTA) for staining.

When analysing lymphocytes, an LN or a spleen was put in a 60 mm × 15 mm Petri dish containing 2 ml FACS buffer and was ground between two frosted microscope slides. When analysing DCs, an LN or a spleen was digested as above. Cell suspensions were filtered through a 70-μm filter and spun for 5 min at 500*g*. Then, the cell pellets were resuspended in FACS buffer for staining.

### Flow cytometry

Preparation of single-cell suspensions from spleens, LNs and brains is described above. Nonspecific binding was blocked using an Fc receptor-blocking solution (TruStain FcX, BioLegend, 101320, 1:200) for 10 min at 4 °C before immunostaining. Subsequently, the cells were stained with corresponding antibodies for 30 min at 4 °C. Then, cells were washed to remove excess antibodies and resuspended in FACS buffer. Samples were run on an Attune NxT flow cytometer and then analysed using FlowJo software (10.8.1, Tree Star).

### Enzyme-linked immunosorbent assay

CSF and serum were collected from mice as previously described^9^. The serum and CSF were then diluted with PBS containing 0.1% BSA in a 1:1 ratio. Plates (96-well) were coated with 100 μl of heat-inactivated or PFA-inactivated purified HSV-2 (10^4^ to 10^5^ plaque-forming units equivalent per 100 μl) for virus-specific immunoglobulin measurement or goat anti-mouse immunoglobulin (SouthernBiotech, 1010-01, 1:1,000) and then incubated overnight at 4 °C. These plates were then washed with PBS–Tween 20 and blocked for 2 h with 5% FBS in PBS. Samples were then plated in the wells and incubated for at least 4 h at room temperature. After being washed in PBS–Tween 20, HRP-conjugated anti-mouse immunoglobulin antibodies (SouthernBiotech, 1010-05, 1:5,000) were added in the wells for 1 h, followed by washing and addition of TMB solution (eBioscience). Reactions were stopped with 1 N H_2_SO_4_, and absorbance was measured at 450 nm. The total antibody titres were defined by using an immunoglobulin standard (C57BL/6 mouse immunoglobulin panel; SouthernBiotech).

### Western blot

rAAV-RFP-infected retinas or control retinas were digested with cocktail containing 1 mg ml^−1^ collagenase D (Roche) and 30 μg ml^−1^ DNase I (Sigma-Aldrich) in RPMI at 37 °C for 45 min. Tissues were pipetted to break tissue down and filtered through a 70-μm filter. Then, cells were lysed in RIPA buffer and boiled for 5 min with sample buffer. Western blotting was carried out in a similar manner to that previously reported^9^. In brief, 15% gels were used and run at 10 mA per gel for 30 min and 40 mA per gel until ladder separation. Wet transfer was carried out at 120 mA per gel for 90 min on ice. RFP-Tag rabbit polyclonal antibodies (OriGene Technologies, catalogue no. AP09229PU-N) were used at a concentration of 1:1,000 and incubated overnight in a cold room. After being washed, HRP-conjugated anti-rabbit IgG secondary antibodies (Thermo Fisher, G-21234) were used at a concentration of 1:5,000 at room temperature for 2 h and imaged using the ChemiDoc MP imaging system (Bio-Rad).

### ELISpot assay

Mice were injected with rAAV-RFP through the IVT or AC route. Their dCLNs, sCLNs and retinas were collected 10 days later. Single-cell suspensions were prepared and co-cultured with splenocytes at a ratio of 1:5 with the presence of rAAV-RFP virus peptides (SNYNKSVNV and NGRDSLVNPGPAMAS). rAAV-specific immune responses were quantified using an ELISpot assay (mouse IFNγ ELISpot Kit; R&D, catalogue no. EL485), following the manufacturer’s instructions for the assay.

### Flank tumour inoculation and treatment

Mice were anaesthetized using a mixture of ketamine (50 mg kg^−1^) and xylazine (5 mg kg^−1^), and the flank was shaved and disinfected. A 1-ml syringe with a 30-G needle was used to deliver 100 μl of 500,000 B16 cells subcutaneously. Then, mice were treated with irradiated B16 cells (250,000 cells) through s.c., i.c., AC or IVT administration routes (day 7) along with anti-PD1 (RMP1-14) antibodies (days 7, 9 and 11) through the i.p. route, and their survival was monitored.

### Adoptive transfer

To directly analyse the immune response (antigen-specific T and B cells) in dCLNs and sCLNs after IVT or AC injection, we transferred OT-II and B1-8 cells and analysed their response after different immunization routes.

To analyse antigen-specific B cell response, we followed a previously reported method^45^. In brief, CD45.2 C57BL/6 recipient mice (6–8 weeks of age) were primed by i.p. immunization with 50 mg of OVA (Sigma, A5503) precipitated in alum at a 2:1 ratio in PBS. Two weeks later, resting B cells were isolated from CD45.1.2 B1-8 mice with a mouse B cell isolation kit (Stemcell, 19854). Then, the B cells were labelled with CellTrace Violet Cell Proliferation Kit (Thermo Fisher, C34557). A total of 5 million cells were transferred intravenously into recipient mice. Eight hours later, the mice were immunized with 10 μg NP20–OVA (Biosearchtech, N-5051) through IVT or AC injection. Igλ^+^ B1-8^hi^ cell proliferation and germinal centre formation in dCLNs and sCLNs were analysed at day 7.

For antigen-specific T cell response, OVA-specific CD4^+^ T cells were isolated from CD45.1 OT-II mice with mouse CD4^+^ T Cell Isolation Kit (Stemcell, 19852). Then, the CD4^+^ T cells were labelled using the CFSE Cell Proliferation Kit (Thermo Fisher, C34554). A total of 5 million cells were transferred intravenously into CD45.2 recipient mice. Eighteen hours later, the mice were immunized with 2 μl 50 μg OVA (Sigma, A5503) plus 1 μg poly(I:C) (Invivogen, tlrl-picw) through IVT or AC injection. A 150 μg quantity of FTY720 (Sigma, SML0700) was i.p. injected to inhibit the circulation of primed T cells 24 h after immunization^46^. OVA-specific CD4^+^ T cell proliferation in ingLNs, dCLNs and sCLNs was analysed 72 h after immunization.

### IVT, AC, i.c. and ICM injection

Mice aged 6–10 weeks old were anaesthetized through i.p. injection of a mixture of ketamine (50 mg kg^−1^) and xylazine (5 mg kg^−1^). For IVT or AC injection, the eye was dilated with tropicamide ophthalmic solution. For IVT injection, a 30-G needle was used to puncture a hole at 1 mm posterior to the corneoscleral junction. A blunt-ended Hamilton syringe with 1 μl of dye, HSV-1, HSV-2, *S. pneumoniae* or irradiated tumour cells was inserted into the vitreous humour about 1–2 mm deep and administered at a rate of 1 μl min^−1^. For AC injection, the hole was punctured close to the corneoscleral junction and a blunt-ended Hamilton syringe was inserted into the AC about 1 to 2 mm deep. After IVT or AC injection, petrolatum ophthalmic ointment was applied on the eye to prevent cataract formation. The method for i.c. injection was similar to that for tumour inoculation, but 3 μl of HSV-1, HSV-2, *S. pneumoniae* or irradiated tumour cells was administered. ICM injection was carried out as previously described^9^. The mice were kept on heating pads and continuously monitored until recovery after the injection.

### Virus and bacteria immunization and challenge

Mice were anaesthetized with a mixture of ketamine (50 mg kg^−1^) and xylazine (5 mg kg^−1^). For HSV immunization, 10^6^ plaque-forming units of heat-inactivated HSV-1 or HSV-2 were AC, IVT, i.p. or i.c. injected. Thirty days later, these mice were challenged through the i.c. route with 10^5^ HSV-1 or HSV-2 and their survival was monitored. For some experiments, CD4^+^ T cell-depleting antibodies (BioXCell, GK1.5, No. BE0003-1, 200 μg for 3 days) were injected before rechallenge. Similar experiments were carried out with *S.* *pneumoniae*; the immunization dose was 10^4^ of heat-inactivated bacteria and the challenge dose was 10^4^ of live bacteria.

### Imaging and quantification of tracer transport

For quantification of fluorescence intensity in the eye or LNs, dextran conjugated to either FITC (40 kDa and 70 kDa) or tetramethylrhodamine (40 kDa and 70 kDa) was injected into the AC or vitreous humour through AC or IVT injection, respectively. To analyse the kinetics of dye drainage from the eye, the eye was collected at serial time points and was homogenized in 150 μl PBS using the bead beating method (Lysing Matrix D, 116913500, MP Biomedicals). Then, homogenized tissue was centrifuged at 10,000*g* for 10 min and 100 μl supernatant was collect into a 96-well plate and fluorescence intensity was read with emission and excitation wavelengths of 494 nm and 514 nm or 555 nm and 585 nm. To measure the dye drainage into LNs, sCLNs and dCLNs were collected 12 h after dye injection. The fluorescence intensity was measured as above.

For measuring nanoparticle draining in the serum, we used a previously published protocol^17^. In brief, serum from mice was isolated and placed on microscope slides to allow small-volume high-sensitivity detection of tracer transport in the blood.

For IVIS imaging of tracer transport, the eye, sCLNs and dCLNs were collected after dye injection through either an AC or IVT administration route. They were imaged using the IVIS Spectrum In Vivo Imaging System (PerkinElmer).

For IVT tracer transport in vivo, 0.2 μl AF647–OVA (2 mg ml^−1^, Thermo Fisher, O34784) was IVT injected into the vitreous humour. A 100 μl volume of sodium fluorescein (1 mg ml^−1^, Santa Cruz, sc-206026) was injected into blood to label blood vessels. The kinetics of AF647–OVA drainage was tracked with the Phoenix MICRON IV imaging microscope.

For analysing the co-localization of optic nerve sheath lymphatics with IVT tracer, 1 μg anti-mouse LYVE1 antibody (R&D, MAB2125) was IVT injected into the vitreous humour. Optic nerve sheaths were collected 2 h later and stained as above.

For tracking the kinetics of dye draining in the eye, 1 μl AF647–OVA (AF647–OVA (2 mg ml^−1^; Thermo Fisher, O34784) was IVT injected into the vitreous humour. The eyes were enucleated at the indicated time points and processed in a manner similar to that previously reported^47^. In brief, eyes were fixed in Hartman’s fixation buffer, and three windows on the eye were created using the previously described window technique^47^. Serial 10-μm sections were obtained using a cryostat (Leica CM190) following dehydration of the tissue in a sucrose gradient up to 30% sucrose. The sections were mounted with ProLong Gold Antifade Mountant with DAPI (Invitrogen, P36931) and imaged with a Leica confocal microscope (Stellaris 8).

### CSF collection

For CSF collection, mice were anaesthetized through i.p. injection of a mixture of ketamine (50 mg kg^−1^) and xylazine (5 mg kg^−1^). The dorsal neck was shaved and sterilized. A 1-cm incision was made at the base of the skull, and the dorsal neck muscles were separated using forceps to expose the cisterna magna. A custom-pulled micropipette (0.75/1 1brl GF; Stoelting) was used to penetrate the dura to collect CSF.

### Brain tumour inoculation and IVIS imaging

Tumour inoculation was carried out as previously described with slight modifications^9^. Mice were anaesthetized through i.p. injection using a mixture of ketamine (50 mg kg^−1^) and xylazine (5 mg kg^−1^). Mouse heads were shaved and scalps were sterilized. A midline scalp incision was made and a burr hole was drilled 2 mm lateral to the sagittal suture and 0.5 mm posterior to the bregma with a 25-G needle. Then, mice were placed in a stereotaxic frame. A 10-μl Hamilton syringe loaded with 3 μl GL261–Luc cells (10^5^ cells) was inserted into the burr hole at a depth of 4 mm from the surface of the brain and left to equilibrate for 1 min before infusion. A micro-infusion pump (World Precision) was used to infuse at 1 μl min^−1^. The syringe was left in place for another minute after the infusion was finished. The skin was stapled and cleaned. Following intramuscular administration of an analgesic (meloxicam and buprenorphine, 1 mg kg^−1^), mice were placed in a heated cage until full recovery. We tracked tumour size weekly thorough IVIS imaging. Mice were anaesthetized using isoflurane and injected through the i.p. route with d-luciferin potassium salt bioluminescent substrate (PerkinElmer, 122799, 200 μl, 30 mg ml−1). After 10 min, mice were imaged using the IVIS Spectrum In Vivo Imaging System (PerkinElmer).

### Preparation of tissue for analysis of CT2A–BFP tumour antigen drainage

i.c. CT2A–BFP tumours were analysed 14 days after injection. Tumours, meninges, IngLNs, dCLNs and sCLNs were collected. Mononuclear cells were isolated and stained.

### Parabiosis

Parabiosis was carried out as previously described with slight modifications^3^. Naive or IVT-immunized C57BL/6 mice of similar age and weight were anaesthetized with a mixture of ketamine (50 mg kg^−1^) and xylazine (5 mg kg^−1^). After shaving the corresponding lateral aspects of each mouse, the skin was cleaned and sterilized with an alcohol prep pad and Betadine surgical scrub. Matching skin incisions were made from just above the knee upwards to the olecranon, and two mice were sutured together with Ethicon 5-0 coated Vicryl absorbable sutures. Then, the skin was stapled and Neosporin + Pain Relief Ointment was applied on the incisions. During the surgery, mice were kept on heating pads and continuously monitored until recovery.

### Ligation of sCLNs or dCLNs

For ligation of LNs, mice were anaesthetized with a mixture of ketamine (50 mg kg^−1^) and xylazine (5 mg kg^−1^) and the rostral neck was shaved and sterilized. A 2-cm incision was made, and the sCLNs and dCLNs were sequentially exposed using forceps. Their afferent lymph vessels were cauterized or kept intact on the basis of the experiment conditions. Then, the incision was closed with a 5-0 Vicryl suture, and mice were subjected to the same postoperative procedures as above.

### rAAV transduction and imaging

Wild-type mice were IVT injected with rAAV (3 × 10^11^ viral genomes) with PBS, VEGFC (1 μg) or sVEGFR3 (1 μg). Then, 1 month later, these mice were rechallenged with rAAV-RFP (3 × 10^11^ viral genomes). Eyes were either imaged on the Phoenix Micron IV or collected 1 month after rAAV-RFP transduction and fixed with 1% PFA overnight at 4 °C. The retina whole mount was carefully dissected and imaged with a Leica confocal microscope (Stellaris 8).

### Three-dimensional imaging of solvent-cleared organs

iDISCO was carried out as previously described (http://www.idisco.info)^22^. The following antibodies were used: goat anti-mouse VEGFR3 (R&D, No. AF743,1:400), rat anti-mouse LYVE1 (R&D, MAB2125,1:400), rabbit anti-mouse LYVE1 (AngioBio, No. 11-034,1:200), mouse anti-human VEGFR3 (Santa Cruz Biotechnology, SC-28297, 1:200), rabbit anti-human LYVE1 (Angio-Proteomie, 102-PA50S, 1:200), goat anti-mouse IgG–AF647 (Invitrogen, A21235, 1:500), donkey anti-goat IgG–AF647 (Invitrogen, A21447, 1:500), goat anti-rabbit IgG–AF555 (Invitrogen, A21428, 1:500). Subsequently, the transparent optic nerves with optic nerve sheaths were imaged using a Leica confocal microscope (Stellaris 8). Three-dimensional rendering was completed using Imaris 8 software (Oxford Instruments).

### Spatial transcriptomics

The 10X Visium Spatial Gene Expression for FFPE slide (PN-1000185) and associated protocols (CG000483) from 10X Genomics were used. Mouse eyeballs were fixed^47^, processed and sectioned^48^ as previously described. Transverse sections of 5 μm in thickness of eyeball were cut using a microtome (RM2255, Leica Biosystems) and carefully placed within the fiducial frame on the Visium slide, and then sections were air dried at room temperature overnight and stored in a desiccant container before spatial transcriptomics experiment. The FFPE sections were baked, stained with haematoxylin–eosin and then imaged using a Keyence bz-x800 all-in-one fluorescence microscope. Then, cell permeabilization and library preparation was carried out following the Visium Spatial Gene Expression FFPE User Guide using the supplied reagents (10X Genomics). The generated libraries were sequenced and analysed using Space Ranger (version 2.1.0), and data were analysed using Seurat 4.9.9.9040.

### Image processing and analysis

Quantitative analysis of rAAV-infected cells was carried out using either FIJI or ImageJ image-processing software (NIH or Bethesda).

### Statistical analysis

All statistical analyses were carried out using GraphPad Prism software. Data were analysed with a two-tailed unpaired Student’s *t*-test or a one-way ANOVA with multiple comparisons testing (Dunnett) with Prism software. Statistical significance is defined as *P < 0.05, **P < 0.01 and ***P < 0.001.

### Reporting summary

Further information on research design is available in the Nature Portfolio Reporting Summary linked to this article.

## Online content

Any methods, additional references, Nature Portfolio reporting summaries, source data, extended data, supplementary information, acknowledgements, peer review information; details of author contributions and competing interests; and statements of data and code availability are available at 10.1038/s41586-024-07130-8.

### Supplementary information

Reporting Summary

### Source data

Source Data Figs. 1–4 and Extended Data Figs. 1–3 and 8–10

## Data Availability

The spatial transcriptomics dataset of the mouse eye and optic nerve is publicly available at the National Center for Biotechnology Information Gene Expression Omnibus under accession number PRJNA1046563. All datasets generated and/or analysed during the current study are available in the article. Source data are provided with this paper.

## Code availability

All of the code used for analysis is described in the Methods. Detailed files will be made available upon on request.
