# Supplementary material for: Compartmentalized ocular lymphatic system mediates eye–brain immunity
Source: Nature. 2024 Feb 28;628(8006):204–11. doi: 10.1038/s41586-024-07130-8 (PMC10990932; doi:10.1038/s41586-024-07130-8)
Supplement: Supplementary file 1 — Reporting Summary [file 41586_2024_7130_MOESM1_ESM.pdf]

nature portfolio

Corresponding author(s): Eric Song

Last updated by author(s): December 13 2023

Reporting Summary

Nature Portfolio wishes to improve the reproducibility of the work that we publish. This form provides structure for consistency and transparency in reporting. For further information on Nature Portfolio policies, see our Editorial Policies and the Editorial Policy Checklist.

Statistics

For all statistical analyses, confirm that the following items are present in the figure legend, table legend, main text, or Methods section.

n/a	Confirmed	
☐	☒	The exact sample size (*n*) for each experimental group/condition, given as a discrete number and unit of measurement
☐	☒	A statement on whether measurements were taken from distinct samples or whether the same sample was measured repeatedly
☐	☒	The statistical test(s) used AND whether they are one- or two-sided<br>*Only common tests should be described solely by name; describe more complex techniques in the Methods section.*
☐	☒	A description of all covariates tested
☐	☒	A description of any assumptions or corrections, such as tests of normality and adjustment for multiple comparisons
☐	☒	A full description of the statistical parameters including central tendency (e.g. means) or other basic estimates (e.g. regression coefficient) AND variation (e.g. standard deviation) or associated estimates of uncertainty (e.g. confidence intervals)
☐	☒	For null hypothesis testing, the test statistic (e.g. *F*, *t*, *r*) with confidence intervals, effect sizes, degrees of freedom and *P* value noted<br>*Give P values as exact values whenever suitable.*
☒	☐	For Bayesian analysis, information on the choice of priors and Markov chain Monte Carlo settings
☒	☐	For hierarchical and complex designs, identification of the appropriate level for tests and full reporting of outcomes
☒	☐	Estimates of effect sizes (e.g. Cohen's *d*, Pearson's *r*), indicating how they were calculated

Our web collection on statistics for biologists contains articles on many of the points above.

Software and code

Policy information about availability of computer code

Data collection	No software was used
Data analysis	R studio, Seurat 4.9.9.9040, PRISM 8.1.2, excel 16.16.6, FIJI (ImageJ) 2.0.0

For manuscripts utilizing custom algorithms or software that are central to the research but not yet described in published literature, software must be made available to editors and reviewers. We strongly encourage code deposition in a community repository (e.g. GitHub). See the Nature Portfolio guidelines for submitting code & software for further information.

Data

Policy information about availability of data

All manuscripts must include a data availability statement. This statement should provide the following information, where applicable:

- Accession codes, unique identifiers, or web links for publicly available datasets
- A description of any restrictions on data availability
- For clinical datasets or third party data, please ensure that the statement adheres to our policy

All data is available with the manuscript

Research involving human participants, their data, or biological material

Policy information about studies with human participants or human data. See also policy information about sex, gender (identity/presentation), and sexual orientation and race, ethnicity and racism.

Reporting on sex and gender	The study did not involve human participants
Reporting on race, ethnicity, or other socially relevant groupings	The study did not involve human participants
Population characteristics	The study did not involve human participants
Recruitment	The study did not involve human participants
Ethics oversight	The study did not involve human participants

Note that full information on the approval of the study protocol must also be provided in the manuscript.

Field-specific reporting

Please select the one below that is the best fit for your research. If you are not sure, read the appropriate sections before making your selection.

☒ Life sciences ☐ Behavioural & social sciences ☐ Ecological, evolutionary & environmental sciences

For a reference copy of the document with all sections, see nature.com/documents/nr-reporting-summary-flat.pdf

Life sciences study design

All studies must disclose on these points even when the disclosure is negative.

Sample size	Sample sizes of all experiments are reported,. We graphed a summary of key phenotypes we observed throughout multiple experiments. With these two experiments showing ~70%<br>response rates, the sample size calculation required for an alpha of 0.05 is N = 6 (power 95%) and N = 5 (power 90%).
Data exclusions	Data was only excluded when experiment collection machine failed and no data points were collected. as were not able to produce readable result files
Replication	All experiments were repeated as a variation (changes in time points) or in smaller n numbers, and showed similar trends and experimental conclusions. For most experiments depicted, they are concatenated examples of all experiments performed (not true for experiments where variation between different days are higher, ie. MFI measurements). All experiments were reproducible. All experiments were replicated at least twice and reported accordingly in the legends
Randomization	Mice were randomized to each group and experiments were controlled with controls in each day even for replication experiments
Blinding	At least one investigator was blinded for each study to allow for non-biased reporting of data

Reporting for specific materials, systems and methods

We require information from authors about some types of materials, experimental systems and methods used in many studies. Here, indicate whether each material, system or method listed is relevant to your study. If you are not sure if a list item applies to your research, read the appropriate section before selecting a response.

Materials & experimental systems

n/a	Involved in the study	
☐	☒	Antibodies
☐	☒	Eukaryotic cell lines
☒	☐	Palaeontology and archaeology
☐	☒	Animals and other organisms
☒	☐	Clinical data
☒	☐	Dual use research of concern
☒	☐	Plants

Methods

n/a	Involved in the study	
☒	☐	ChIP-seq
☐	☒	Flow cytometry
☒	☐	MRI-based neuroimaging

Antibodies

Antibodies used

Anti-CD3 (145-2C11, APC, 152306), Anti-CD4 (RM4-5, PerCP, 100538; RM4-5, BV605, 100548), Anti-CD8α (53-6.7, BV605, 100744; 53-6.7, BV785, 100750), anti-CD11b (M1/70, BV711, 101242), anti-CD19 (6D5, APC-Cy7, 115530), anti-IA/IE (M5/114.15.2, AF488, 107616), anti-CD44 (IM7, AF700, 103026; BV421, 103040), anti-CD45 (30-F11, APC-Cy7, 103116), anti-CD45.1 (A20, BV785, 110743), anti-CD45.2 (104, Pacific Blue, 109820), anti-CD64 (X54-5/7.1, PE, 139304), anti-CD95 (Jo2, PE-Cy7, 557653), anti-B220 (Ra3-6B2, AF700, 103232), anti- GL7 (GL7, FITC, 144603), anti-NK1.1 (PK136, APC-Cy7, 108724), anti- TCRβ (H57-597, APC-Cy7, 109220) were purchased from BD Biosciences or BioLegend. Anti-Ig λ Light Chain, (JC5-1, FITC, 130-098-415) was purchased from Miltenyibiotec.

Goat anti-mouse VEGFR3 (#AF743) and Rat-anti-mouse LYVE1 (# MAB2125) were purchased from R&D. Mouse anti-human VEGFR3 (SC-28297) were purchased from Santa Cruz Biotechnology. Rabbit anti-Prox1 (11-002P) and Rabbit anti Human LYVE-1 (102-PA50S) were purchased from Angio-proteomie. Podoplanin (127402) were purchased from biolegend. Armenian Hamster anti- mouse CD31 (2H8) were purchased from Gene Tex. Goat anti-mouse IgG-AF647 (A21235), Donkey anti-goat IgG- AF647 (A21447), Goat anti-rabbit IgG-AF555 (A21428). Goat anti-Armenian Hamster IgG- AF-488 (# A78963), Goat anti-Syrian Hamster IgG-AF-488 (# A78958) were purchased from Invitrogen.
RFP-Tag rabbit polyclonal antibody (#AP09229PU-N) were purchased from OriGene Technologies.
Mouse anti- Zebrafish zns-2 (ZDB-ATB-081002-34) were purchased from ZIRC. Chicken anti-GFP (Cat# GFP-1010) were purchased from Aves. AF488 conjugated goat anti-mouse IgG AF488 (115-545-146) and Cy5-conjugated donkey anti-chicken IgY (703-175-155) were purchased from Jackson Immuno Research.
Goat anti-mouse immunoglobulin (1010-01) and HRP-conjugated anti-mouse Ig antibodies (1010-05) were purchased from SouthernBiotech.
Rat anti-mouse CD4 antibody (#BE0003-1, GK1.5) were purchased from BioXCell.

Validation

All the antibodies are commonly used and validated antibodies per manufacturer instructions. Please refer to each manufacturer as listed above. No antibodies used were newly generated or unconventionally used antibodies.

Eukaryotic cell lines

Policy information about cell lines and Sex and Gender in Research

Cell line source(s)

GL261-Luc cells were a gift from J. Zhou (Yale Neurosurgery) and were cultured in RPMI supplemented with 10% FBS, 1% penicillin/streptomycin and 1% sodium pyruvate. CT-2A-BFP cells were a gift from T. Mathivet (Paris Centre de Recherche Cardiovasculaire). B16 cells were a gift from N. Palm (Yale Immunobiology).

Authentication

Cells were not authenticated separately, but phenotypes were validated with similar authenticated cell lines from the NIH.

Mycoplasma contamination

All Cells tested negative for mycoplasma contamination

Commonly misidentified lines
(See ICLAC register)

The study did not use commonly misidentified lines

Animals and other research organisms

Policy information about studies involving animals; ARRIVE guidelines recommended for reporting animal research, and Sex and Gender in Research

Laboratory animals

Six-to-ten-week-old mixed sex C57BL/6 mice, B6.Cg-Tg(TcraTcrb)425Cbn/J (OT-II), B6.129P2(C)-Ightm2Cgn/J (B1-8) and B6.129S2-IghtmICgn/J (μMT) mice were purchased from Jackson Laboratory and Charles River and subsequently bred and housed at Yale University. PROX1CreERT2;CDH5 Dre;R26-STOP-mCherry and VEGFR3-CreERT2; R26-MTMG mice were gifts from the Thomas lab. All procedures used in this study (sex-matched, age-matched) complied with federal guidelines and the institutional policies of the Yale School of Medicine Animal Care and Use Committee

Wild animals

The study did not involve wild animals

Reporting on sex

Mixed sex mice were used for our studies ans we did not see any sex-biased phenotypes

Field-collected samples

The study did not involve samples collected from the field

Ethics oversight

All animal work was approved by Yale Institutional Animal Care & Use Committee

Note that full information on the approval of the study protocol must also be provided in the manuscript.

Plants

Seed stocks	The study did not involve plants
Novel plant genotypes	The study did not involve plants
Authentication	The study did not involve plants

Flow Cytometry

Plots

Confirm that:

☒ The axis labels state the marker and fluorochrome used (e.g. CD4-FITC).

☒ The axis scales are clearly visible. Include numbers along axes only for bottom left plot of group (a 'group' is an analysis of identical markers).

☒ All plots are contour plots with outliers or pseudocolor plots.

☒ A numerical value for number of cells or percentage (with statistics) is provided.

Methodology

Sample preparation

For brain tissues, tissues were collected and incubated in a digestion cocktail containing 1 mg ml−1 collagenase D (Roche) and 30 μg ml−1 DNase I (Sigma-Aldrich) in RPMI at 37 °C for 45 min. Tissues were pipetted to break tissue down and filtered through a 70-μm filter. Then, cells were mixed in 3 ml of 25% Percoll (Sigma-Aldrich) solution and centrifuged at 580g for 15 min without brake. The Percoll layer was removed, and cell pellets were treated with 0.5ml ACK buffer and spun for 5 minutes at 500g. Then the cell pellets were resuspended in FACS buffer (PBS +2% FBS+ 1mM EDTA) for staining.

For LN or spleen was put in a 60-mm x15-mm petri dish containing 2 mL FACS buffer and was ground between 2 frosted microscope slides. When analyzing DCs, a LN or spleen were digested as above. Cell suspension was filtered through a 70-μm filter and spun for 5 minutes at 500g. Then the cell pellets were resuspended in FACS buffer for staining.

Instrument

Preparation of single-cell suspensions from spleen, LNs and brains are described above. Nonspecific binding was blocked using a Fc receptor-blocking solution (TruStain FcX™, 101320, BioLegend) for 10 minutes at 4°C prior to immunostaining. Subsequently, the cells were stained with corresponding antibodies for 30 min at 4°C. Then, cells were washed to remove excess antibodies and resuspended in FACS buffer. Samples were run on an Attune NxT flow cytometer and then analyzed using FlowJo software (10.8.1, Tree Star).

Software	FlowJo software (10.8.1, Tree Star)
Cell population abundance	No sorts were performed
Gating strategy	Please refer to supplemental figures and methods for detailed gating strategies

☒ Tick this box to confirm that a figure exemplifying the gating strategy is provided in the Supplementary Information.
